# Theoretical Investigation of Single-Atom Catalysts for Hydrogen Evolution Reaction Based on Two-Dimensional Tetragonal V_2_C_2_ and V_3_C_3_

**DOI:** 10.3390/ma18050931

**Published:** 2025-02-20

**Authors:** Bo Xue, Qingfeng Zeng, Shuyin Yu, Kehe Su

**Affiliations:** 1 School of Physical Science and Technology, Northwestern Polytechnical University, Xi’an 710129, China; 2 MSEA International Institute for Materials Genome, Langfang 065500, China; zengqf@dianyunkeji.com (Q.Z.); yusy@dianyunkeji.com (S.Y.); 3 Particle Cloud Biotechnology (Hangzhou) Co., Ltd., Hangzhou 310018, China; 4 Science and Technology on Thermostructural Composite Materials Laboratory, Northwestern Polytechnical University, Xi’an 710072, China; 5 School of Chemistry and Chemical Engineering, Northwestern Polytechnical University, Xi’an 710129, China

**Keywords:** transition metal carbide, single-atom catalysts, hydrogen evolution reaction, first-principles computations

## Abstract

Developing stable and effective catalysts for the hydrogen evolution reaction (HER) has been a long-standing pursuit. In this work, we propose a series of single-atom catalysts (SACs) by importing transition-metal atoms into the carbon and vanadium vacancies of tetragonal V_2_C_2_ and V_3_C_3_ slabs, where the transition-metal atoms refer to Ti, V, Cr, Mn, Fe, Co, Ni, and Cu. By means of first-principles computations, the possibility of applying these SACs in HER catalysis was investigated. All the SACs are conductive, which is favorable to charge transfer during HER. The Gibbs free energy change (Δ*G*_H*_) during hydrogen adsorption was adopted to assess their catalytic ability. For the V_2_C_2_-based SACs with V, Cr, Mn, Fe, Ni, and Cu located at the carbon vacancy, excellent HER catalytic performance was achieved, with a |Δ*G*_H*_| smaller than 0.2 eV. Among the V_3_C_3_-based SACs, apart from the SAC with Mn located at the carbon vacancy, all the SACs can act as outstanding HER catalysts. According to the Δ*G*_H*_, these excellent V_2_C_2_- and V_3_C_3_-based SACs are comparable to the best-known Pt-based HER catalysts. However, it should be noted that the V_2_C_2_ and V_3_C_3_ slabs have not been successfully synthesized in the laboratory, leading to a pure investigation without practical application in this work.

## 1. Introduction

Energy is an important issue related to the economically and environmentally sustainable development of the world [1,2]. The conventional sources of energy, such as coal, petroleum, and natural gas, are available in limited quantity and not renewable and environmentally friendly [3,4]. To alleviate the looming energy crises, there is a global drive to explore and develop renewable and clean alternatives to fossil fuels [5]. This pressing need has sparked significant scientific efforts aimed at developing sustainable energy technologies to satisfy the growing energy demands of society while reducing carbon emissions as well as environmental degradation. As a clean and sustainable energy carrier, hydrogen (H_2_) possesses high gravimetric energy density and emits only water as a byproduct when utilized in a fuel cell [6]. Hydrogen production driven by renewable energy sources has become a promising strategy for generating carbon-free fuel from sustainable energy at low cost.

Electrochemical water splitting on the cathode is an efficient approach for hydrogen production, where the hydrogen evolution reaction (HER) plays a key role in producing H_2_ [7]. During the HER process, highly effective catalysts are required to reduce the energy barrier and boost H_2_ production. Among the various types of electrocatalysts, Pt-based catalysts have demonstrated exceptional electrocatalytic performance and have been acknowledged as the most effective HER catalysts [8,9]. However, the limitation in availability and the high cost of Pt obstruct large-scale implementation, which leads to the exploration of alternatives as substitutes for Pt-based catalysts [10,11,12]. Up to now, a variety of efficient catalysts based on abundant elements have been developed with the advancement of nanotechnology. Zhao et al. successfully synthesized a series of cobalt-doped Ni_0.85_Se chalcogenides (Co*_x_*Ni_0.85-*x*_Se, *x* = 0.05, 0.1, 0.2, 0.3, and 0.4) as electrocatalysts for HER [13]. The results indicate that Co_0.1_Ni_0.75_Se displays the highest HER performance. When adopting graphene oxide (rGO) in this catalyst as the support, the supported Co_0.1_Ni_0.75_Se exhibits even better performance than unsupported Co_0.1_Ni_0.75_Se, which is determined by the decrease in the HER overpotential of Co_0.1_Ni_0.75_Se/rGO (103 mV) compared to Co_0.1_Ni_0.75_Se (153 mV) at a current density of 10 mA cm^−2^, and the lower Tafel slope (43 mV dec^−1^) and kinetic resistance (21.34 Ω) compared to Co_0.1_Ni_0.75_Se (47 mV dec^−1^, 30.23 Ω). Ito and coworkers reported that N, S co-doped nanoporous graphene possesses high HER catalytic ability at low operating potential, comparable to two-dimensional (2D) MoS_2_ [14]. Wang et al. reported a double-deck carbon-coated V_8_C_7_ network as an efficient HER electrocatalyst. The HER catalytic performances of the V_8_C_7_ network are comparable to those of Pt in all pH conditions, with an overpotential of 38, 77, and 47 mV at a current density of −10 mA cm^−2^ in 0.5 M H_2_SO_4_, 0.1 M phosphate buffer, and 1 M KOH, respectively [15]. The significant progress of non-Pt-based catalysts encourages researchers to explore more noble-metal-free catalytic materials for HER.

Since 2D materials possess special physical and chemical properties, such as a large specific surface area [16], excellent optical transparency [17], and good mechanical properties [18], their potential application in HER catalysis has been intensively studied for decades [19]. Among the various 2D materials, 2D transition-metal carbides (TMCs) have exhibited great potential as electrocatalysts for HER due to their outstanding characteristics, including their excellent structural stability, high electrical conductivity, and large active surface. A class of 2D TMCs known as MXenes show great potential to act as HER catalysts. Bai et al. introduced many applications of MXene-based HER electrocatalysts from theoretical and experimental perspectives [20]. According to their review, many MXene-based materials, such as O-terminated Ti_3_C_2_, nanoribbons of Ti_3_C_2_, transition-metal-promoted V_2_CO_2_, and Co-substituted Mo_2_CT*_x_*, are promising candidates for HER catalysts. In addition to MXenes, many other 2D TMCs have also been reported to possess HER catalytic ability. By performing first-principles calculations, Yu et al. found that NbC_2_, TaC_2_, and MoC_2_ possess excellent HER catalytic ability under the reaction controlled by the Volmer–Heyrovsky mechanism [21]. These studies show that 2D TMCs possess significant potential in catalyzing HER.

With the development of catalyst preparation methods, the rational design of potential catalysts for HER has become fascinating. Among the different types of catalysts, single-atom catalysts (SACs), with only isolated atoms dispersed on the support surface, have attracted considerable interest due to their maximum atom utilization efficiency and high selectivity [22,23,24]. The high dispersion of isolated atoms can greatly reduce the usage of metals, which can greatly lower the application cost of SACs. Importantly, SACs possess unique spatial and electronic structures, and the electronic structure can be adjusted via heteroatom doping, thus achieving a specific coordination environment of the isolated atoms [25]. The active sites in SACs typically consist of individual metal atoms and the coordinating atoms of the support material [26]. Up to now, many species of metal atoms (precious and transition metals) and different support materials have been adopted in SACs with great HER catalytic ability. For example, Qiu et al. reported that isolated nickel atoms anchored to nanoporous graphene exhibit excellent HER catalysis with a low overpotential of about 50 mV and a Tafel slope of 45 mV dec^−1^ in 0.5 M H_2_SO_4_, together with high cycling stability [27]. Zhang and coworkers developed an SAC by importing single Pt atoms into the Mo vacancies of Mo_2_TiC_2_T*_x_* MXene, and this SAC shows excellent HER catalytic performance with low overpotentials of 30 and 77 mV at current densities of 10 and 100 mA cm^−2^, respectively [28]. Furthermore, this Mo_2_TiC_2_T*_x_*-based SAC possesses a mass activity about 40 times greater than that of a Pt-on-carbon catalyst. Deng et al. demonstrated that the HER catalytic ability of the in-plane S atoms of 2D MoS_2_ can be enhanced by Pt atom doping [29]. In order to explore more suitable SACs for HER, it is essential to investigate novel combinations of different support materials and single atoms.

Using experimental methods, researchers can prepare SACs based on 2D materials with isolated transition-metal atoms dispersed on the surface of substrates [30,31]. On the other hand, there are many works that theoretically investigate SACs based on 2D materials [32,33,34]. Inspired by these theoretical studies, in this work, we rationally designed a series of SACs theoretically by embedding various transition-metal atoms in the C and V vacancies of the stable 2D tetragonal vanadium carbides V_2_C_2_ and V_3_C_3_ that were identified by previous structural prediction on V-C phases [35,36], where the transition-metal atoms refer to Ti, V, Cr, Mn, Fe, Co, Ni, and Cu. The thermally, dynamically, and mechanically stable V_2_C_2_ and V_3_C_3_ possess good electrical conductivity, which is favorable to electrocatalysis. In addition, different from most 2D materials, each sub-layer of V_2_C_2_ and V_3_C_3_ has the same coordination for the C/V atom in the *xy* plane, which may result in unique HER catalytic properties. After the construction of V_2_C_2_- and V_3_C_3_-based SACs, the HER catalytic performances of the proposed SACs at different adsorption sites were investigated theoretically.

## 2. Computational Details

All the calculations based on spin-polarized density functional theory (DFT) were carried out with the Vienna ab initio simulation package (VASP 5.4.4) [37,38]. The exchange correlation effect was treated using generalized gradient approximation (GGA) [39] in the Perdew–Burke–Ernzerhof (PBE) form [40]. The kinetic energy cut-off was set as 500 eV, and the convergence criteria for electron self-consistent loop and geometry optimization were set as 10^−5^ eV and 0.02 eV/Å, respectively. A vacuum space larger than 15 Å in the *z* direction was chosen to eliminate spurious interactions between adjacent images. The DFT-D3 scheme [41] was employed to capture the long-range van der Waals interaction between the slabs. The 4 × 4 supercells of the V_2_C_2_ and V_3_C_3_ slabs were used to design SACs, and a 4 × 4 × 1 Gamma-centered *k*-point mesh was utilized to sample the Brillouin zone. In addition, to inspect the thermal stability of the studied SACs, ab initio molecular dynamics (AIMD) simulations were conducted at 600 K with a time step of 2 fs for 10 ps. The AIMD calculations were performed using the Andersen thermostat [42] and *NVT* ensemble.

To evaluate the possibility of forming a single vacancy on the V_2_C_2_/V_3_C_3_ slab, the defect formation energy was calculated via the following equation:

(1) $$E_{\mathrm{form}}(V_{i})=E_{\mathrm{perfect}-V_{i}}-E_{\mathrm{perfect}}+n\mu_{i}$$

where $E_{\mathrm{perfect}-V_{i}}$ and $E_{\mathrm{perfect}}$ are the energies of the V_2_C_2_/V_3_C_3_ slab with and without isolated vacancies, respectively, *μ*_i_ is the chemical potential of type i atom, and *n* is the number of vacancies, which is 1 in this study.

The binding energy of transition-metal atoms (TM = Ti, V, Cr, Mn, Fe, Co, Ni, and Cu) embedded in the vacancies of the V_2_C_2_/V_3_C_3_ slab was determined as follows:

(2) $$E_{\mathrm{bind}}=E_{\mathrm{TM}+S}-E_{S}-E_{\mathrm{TM}}$$

where *E*_TM+S_, *E*_S_, and *E*_TM_ represent the energies of the designed SACs, defective V_2_C_2_/V_3_C_3_ slabs, and a single transition-metal atom in its bulk phase, respectively. According to the defined criteria, more negative values of *E*_bind_ correspond to more stable structures.

It is widely accepted that HER occurs via a multi-step electrochemical process. Specifically, in acidic solutions, the mechanism of the HER process involves hydrogen adsorption (Volmer reaction) followed by hydrogen desorption (Heyrovsky reaction and/or Tafel reaction) [43]:

H^+^ + e^−^ +* → H* (Volmer) (3)

H* + H^+^ + e^−^ → H_2_ + * (Heyrovsky) (4)

H* + H* → H_2_ + * (Tafel) (5)

where the asterisk refers to the adsorption sites on the surfaces of the catalysts. Both Volmer and Heyrovsky/Tafel reactions influence the HER activity, and the Volmer reaction is a dominant step for the overall HER process, which can be applied to assess the HER catalytic ability of the catalyst [44]. The change in Gibbs free energy for hydrogen adsorption (∆*G*_H∗_) during the Volmer step is an effective descriptor for measuring HER catalytic activity and can be obtained by the following expression:

(6) $$\Delta G_{H*}=\Delta E_{H}+\Delta E_{\mathrm{ZPE}}-T\Delta S_{H}$$

In this expression, Δ*E*_H_ is the hydrogen adsorption energy and is calculated using the following equation:

(7) $$\Delta E_{H}=E_{\left(\mathrm{catalyst}+H\right)}-E_{\mathrm{catalyst}}-\frac{1}{2}E_{H_{2}}$$

where *E*_catalyst+H_ and *E*_catalyst_ denote the energies of the SACs with and without an adsorbed hydrogen atom, respectively, and $E_{H_{2}}$ is the energy of the hydrogen molecule. Δ*E*_ZPE_ is the zero-point energy (*E*_ZPE_) difference, and the contributions from the substrate to *E*_ZPE_ are negligible. Hence, Δ*E*_ZPE_ is computed by

(8) $$\Delta E_{\mathrm{ZPE}}=E_{\mathrm{ZPE}}^{H}-\frac{1}{2}E_{\mathrm{ZPE}}^{H_{2}}$$

where $E_{\mathrm{ZPE}}^{H}$ and $E_{\mathrm{ZPE}}^{H_{2}}$ are the *E*_ZPE_ of the adsorbed hydrogen atom and H_2_ in the gas phase, respectively. *T* is the temperature (*T* = 298.15 K), and *T*Δ*S*_H_ is the vibrational entropy difference during hydrogen adsorption. It should be noted that the contributions from the substrate to entropy are insignificant. Thus, *T*Δ*S*_H_ can be approximated as the entropy difference between the adsorbed hydrogen atom and hydrogen in a gaseous state. At 298.15 K, the entropy of gaseous H_2_ is approximately 0.40 eV [45].

## 3. Results and Discussion

### 3.1. Structures, Stability, and Active Sites

Pristine 2D V_2_C_2_ and V_3_C_3_ are tetragonal structures, and they possess two and three sub-layers, respectively [36]. For each sub-layer of these two vanadium carbides, each C/V atom is four-fold coordinated with its neighboring V/C atoms in the *xy* plane. For (4 × 4)-V_2_C_2_, the vacancy formation energies (*E*_form_) of C and V vacancies are 8.52 and 10.54 eV, respectively. Given that the surfaces of V_3_C_3_ are more likely to produce vacancy defects compared to the middle sub-layer, the *E*_form_ of the C_surf_ (surface C) and V_surf_ (surface V) vacancies of (4 × 4)-V_3_C_3_ were calculated, and the corresponding *E*_form_ are 7.25 and 10.82 eV, respectively. It is evident that both V_2_C_2_ and V_3_C_3_ show greater potential to form carbon defects on the surface with relatively low *E*_form_ compared to vanadium defects. The binding energies (*E*_bind_) of transition-metal atoms embedded in the vacancies are listed in Table 1 and Table 2 for (4 × 4)-V_2_C_2_ and (4 × 4)-V_3_C_3_, respectively. The *E*_bind_ of transition-metal atoms at the C vacancy of (4 × 4)-V_2_C_2_ range from 0.92 to 2.27 eV, while those at the V vacancy range from −5.02 to −1.14 eV. The *E*_bind_ at the vacancies of (4 × 4)-V_3_C_3_ exhibit a similar range to those of defective (4 × 4)-V_2_C_2_. The Cr atom imported into the carbon defects shows the biggest binding energies (2.27 and 2.90 eV for V_2_C_2_ and V_3_C_3_ supercells, respectively), while the Ti atom in the vanadium defects exhibits the smallest binding energies (−5.02 and −5.61 eV for V_2_C_2_ and V_3_C_3_ supercells, respectively). Although the vanadium defects have higher formation energies compared with the carbon defects, they exhibit negative binding energies for all the imported transition-metal atoms. Taking into account both formation energies and binding energies, both carbon and vanadium defects are considered to be replaced by transition-metal atoms in this study.

**Figure 1:**
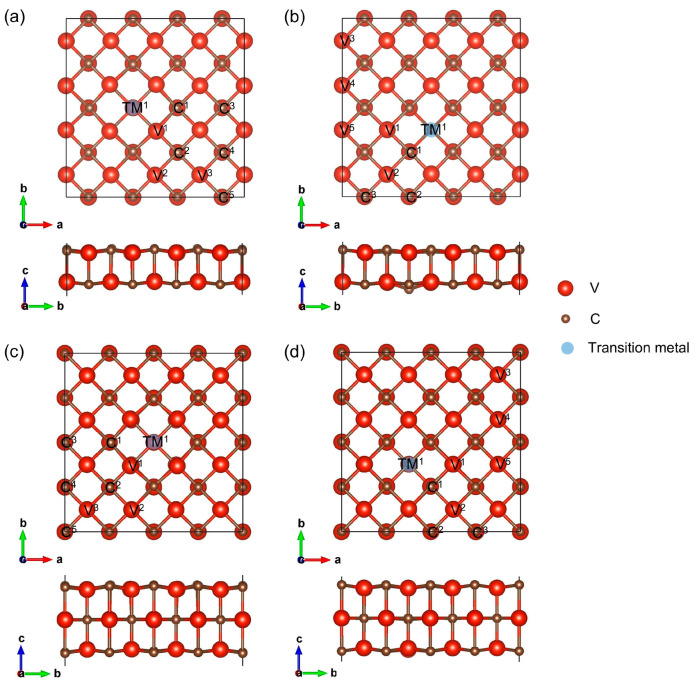
The structures and adsorption sites of (**a**) TM@(4 × 4)-V_2_C_2_-V_C_, (**b**) TM@(4 × 4)-V_2_C_2_-V_V_, (**c**) TM@(4 × 4)-V_2_C_2_-V_surf-C_, and (**d**) TM@(4 × 4)-V_2_C_2_-V_surf-V_. TM atoms are Ti, V, Cr, Mn, Fe, Co, Ni, and Cu for the C/C_surf_ vacancies, while they are Ti, Cr, Mn, Fe, Co, Ni, and Cu for the V/V_surf_ vacancies.

The lattice constants of (4 × 4)-V_2_C_2_ with C and V vacancies are 11.400 and 11.389 Å, respectively, slightly smaller than those of (4 × 4)-V_2_C_2_ (11.415 Å). Figure 1a,b present the configurations of the V_2_C_2_-based SACs, along with the considered adsorption sites for HER, and Figures S1 and S2 (Supplementary Materials) show the relaxed structures of the SACs. It can be seen that there are significant height differences between the substrate and transition-metal atoms located at the C vacancy (Figure S1). This is because the radii of transition-metal atoms are bigger than the radius of a carbon atom, and a C vacancy does not have enough space to accommodate a transition-metal atom. When transition-metal atoms are stabilized at the V vacancy, the encased transition-metal atoms and surface of defective (4 × 4)-V_2_C_2_ are almost at the same height (Figure S2). The SACs are labeled TM@(4 × 4)-V_2_C_2_-V_C_ and TM@(4 × 4)-V_2_C_2_-V_V_, as shown in Figures S1 and S2, respectively. For TM@(4 × 4)-V_2_C_2_-V_C_ and TM@(4 × 4)-V_2_C_2_-V_V_, the anchored transition-metal atoms are surrounded by five V atoms and five C atoms, respectively. In addition, the lattice constants of optimized TM@(4 × 4)-V_2_C_2_-V_C_ and TM@(4 × 4)-V_2_C_2_-V_V_ are listed in Tables S1 and S2 (Supplementary Materials), respectively. The lattice constants of most TM@(4 × 4)-V_2_C_2_-V_C_ are bigger than those of pristine (4 × 4)-V_2_C_2_, while those of most TM@(4 × 4)-V_2_C_2_-V_V_ are smaller than those of (4 × 4)-V_2_C_2_. The lattice constant changes are related to the sizes of the vacancies, the radii of imported transition-metal atoms, and the heights of imported atoms from the substrate. Larger vacancies, smaller radii of imported atoms, and higher anchoring heights are conducive to smaller lattice constants.

The lattice constants of (4 × 4)-V_3_C_3_ with C_surf_ and V_surf_ vacancies are 11.508 and 11.493 Å, respectively, slightly smaller than those of pristine (4 × 4)-V_3_C_3_ (11.510 Å) and larger than those of defective (4 × 4)-V_2_C_2_. Figure 1c,d display the configurations of the V_3_C_3_-based SACs, along with the considered adsorption sites for HER, and Figures S3 and S4 (Supplementary Materials) show the relaxed structures of the SACs. The relative position between the imported transition-metal atoms and substrates is similar to the situation in the V_2_C_2_-based SACs. The V_3_C_3_-based SACs are labeled TM@(4 × 4)-V_3_C_3_-V_surf-C_ and TM@(4 × 4)-V_3_C_3_-V_surf-V_, as shown in Figures S3 and S4, respectively. The lattice constants of all the TM@(4 × 4)-V_3_C_3_-V_surf-C_ are bigger than those of pristine (4 × 4)-V_3_C_3_ (Table S3 of the Supplementary Materials), while those of most TM@(4 × 4)-V_3_C_3_-V_surf-V_ are smaller than those of (4 × 4)-V_3_C_3_ (Table S4 of the Supplementary Materials). Overall, the differences in lattice constants are less than 1%, indicating a good stiffness of the substrates.

The thermal stability of the studied SACs was evaluated by AIMD simulations at 600K with a time step of 2 fs. The AIMD results for the V_2_C_2_- and V_3_C_3_-based SACs are shown in Figures S5–S8 (Supplementary Materials). During the simulations, the energy profiles for each catalyst did not fluctuate violently. Furthermore, the studied systems did not undergo any degradation after the 10 ps AIMD simulations. These results demonstrate that all the designed SACs are thermally stable at high temperature.

The considered adsorption sites for HER are marked in Figure 1. For both the V_2_C_2_- and V_3_C_3_-based SACs, when transition-metal atoms (Ti, V, Cr, Mn, Fe, Co, Ni, and Cu) are anchored at the C/C_surf_ vacancy, the tops of the transition-metal atoms (site TM^1^), V atoms (sites V^1^, V^2^, and V^3^), and C atoms (sites C^1^, C^2^, C^3^, C^4^, and C^5^) are chosen as the adsorption sites for HER. For the SACs with transition-metal atoms (Ti, Cr, Mn, Fe, Co, Ni, and Cu) embedded in the V/V_surf_ vacancy, the nine adsorption sites are the tops of the transition-metal atoms (site TM^1^), C atoms (sites C^1^, C^2^, and C^3^), and V atoms (sites V^1^, V^2^, V^3^, V^4^, and V^5^).

### 3.2. Electronic Conductivity

Electronic conductivity is closely related to the efficiency of charge transfer during the catalytic process [46]. The densities of states (DOSs) of the defective V_2_C_2_/V_3_C_3_ supercells and constructed SACs are displayed in Figures S9–S12 (Supplementary Materials). As shown in Figures S9a and S10a, the V_2_C_2_ slab with a C/V vacancy exhibits good electronic conductivity, with the Fermi level falling into a continuum of energy states. Furthermore, all the TM@(4 × 4)-V_2_C_2_-V_C_ and TM@(4 × 4)-V_2_C_2_-V_V_ also possess excellent conductivity (Figures S9b–i and S10b–h). According to the projected density of states (PDOS), the electronic conductivity of the V_2_C_2_-based SACs is dominated by V-d orbitals near the Fermi level. In addition, the DOSs of the defective (4 × 4)-V_3_C_3_ and V_3_C_3_-based SACs have similar characteristics to the DOSs of the V_2_C_2_-based structures (Figures S11 and S12). Overall, similar to other excellent HER catalysts, such as Mo_2_B_2_-, MoS_2_-, and MXene-based catalysts [32,47,48], all the designed SACs exhibit high electronic conductivity, which can ensure efficient charge transfer in HER.

### 3.3. Hydrogen Evolution Reaction Activity of V_2_C_2_-Based Catalysts

Generally, the HER process can be divided into three states, including the initial state H^+^ + e^−^, the intermediate adsorbed H*, and the final H_2_ state. The Gibbs free energy difference between H* and H_2_ (Δ*G*_H*_) is considered a key descriptor of the HER activity. In this work, the HER catalytic ability of 2D V_2_C_2_- and V_3_C_3_-based SACs at different adsorption sites is determined by Δ*G*_H*_. A smaller absolute value of Δ*G*_H*_ means a better HER performance, and the optimal value of Δ*G*_H*_ is zero. In general, catalysts with a | Δ*G*_H*_| smaller than 0.2 eV are regarded as excellent HER catalysts.

For TM@(4 × 4)-V_2_C_2_-V_C_ and TM@(4 × 4)-V_2_C_2_-V_V_, the calculation details for the free energy differences are listed in Tables S5–S19 (Supplementary Materials), including the energies (*E*), *E*_ZPE_, vibrational entropy (*TS*_H_), Gibbs free energies (*G*), and Δ*G*_H*_ of every V_2_C_2_-based SAC at different active sites. For each TM@(4 × 4)-V_2_C_2_-V_C_, the *E*_ZPE_ value of the catalyst with the hydrogen atom adsorbed on the top of the imported transition-metal atom is smaller than that of its counterparts adsorbing H on the top of the C or V atoms. In addition, almost all the *TS*_H_ values are less than 0.1 eV, much smaller than the *E*_ZPE_ values. Hence, compared with *TS*_H_, *E*_ZPE_ contributes more to the free energy.

The Δ*G*_H*_ values of TM@(4 × 4)-V_2_C_2_-V_C_ at different adsorption sites are listed in Table 3. For each TM@(4 × 4)-V_2_C_2_-V_C_, the Δ*G*_H*_ at adsorption site C^1^ is not higher than that at sites on the top of other C atoms, and the Δ*G*_H*_ at adsorption site V^1^ is smaller than that at sites on the top of other V atoms. Compared with other adsorption sites, C^1^ and V^1^ are closer to the imported transition-metal atoms, and the great impact of the transition-metal atoms on hydrogen adsorption leads to the relatively small Δ*G*_H*_ values. Apart from (Ti and Co)@(4 × 4)-V_2_C_2_-V_C_, other TM@(4 × 4)-V_2_C_2_-V_C_ exhibit high HER catalytic ability, with a |Δ*G*_H*_| smaller than 0.2 eV at some active sites. Specifically, there are up to eight adsorption sites that display great HER catalytic activity on the surface of Mn@(4 × 4)-V_2_C_2_-V_C_, including C^1^, C^2^, C^3^, C^4^, C^5^, V^2^, V^3^, and TM^1^. In particular, the Δ*G*_H*_ of Mn@(4 × 4)-V_2_C_2_-V_C_ at site C^2^ is zero, suggesting the best catalytic performance. (Fe, Ni and Cu)@(4 × 4)-V_2_C_2_-V_C_ have two active sites (V^1^ and TM^1^) with a |Δ*G*_H*_| smaller than 0.2 eV, and (V and Cr)@(4 × 4)-V_2_C_2_-V_C_ have only one active site (TM^1^) possessing excellent catalytic capacity. Among all the V_2_C_2_-based SACs, (Cr, Mn, Fe, and Cu)@(4 × 4)-V_2_C_2_-V_C_ possess active sites with a smaller |Δ*G*_H*_| compared to Pt (0.09 eV) [49]. In addition, for each TM@(4 × 4)-V_2_C_2_-V_C_, the |Δ*G*_H*_| value of some sites is lower than that of Mo_2_B_2_ (0.37 eV) [32], and the |Δ*G*_H*_| value of all the sites is lower than that of MoS_2_ (2.0 eV) [50]. It should be noted that pristine tetragonal V_2_C_2_ is not suitable for HER catalysis [36], and the construction of SACs based on tetragonal V_2_C_2_ has enhanced its HER catalytic ability. Besides V_2_C_2_, the HER catalytic performance of Mo_2_B_2_, graphene, and MoS_2_ has also been greatly improved after importing transition-metal atoms [32,34,50], indicating the huge potential of SACs in HER catalysis.

Table 4 lists the Δ*G*_H*_ values of TM@(4 × 4)-V_2_C_2_-V_V_ at different adsorption sites. Similar to TM@(4 × 4)-V_2_C_2_-V_C_, adsorption site C^1^ exhibits a relatively low Δ*G*_H*_ among the sites on the top of the C atoms. Overall, the Δ*G*_H*_ values of sites on the top of the C atoms are lower than those of sites on the top of the V atoms. For each TM@(4 × 4)-V_2_C_2_-V_V_, the free energy differences of all the considered adsorption sites are larger than 0.3 eV, suggesting inefficient HER catalytic performance.

### 3.4. Hydrogen Evolution Reaction Activity of V_3_C_3_-Based Catalysts

For TM@(4 × 4)-V_3_C_3_-V_surf-C_ and TM@(4 × 4)-V_3_C_3_-V_surf-V_, the calculation details for the free energy differences are listed in Tables S20–S34 (Supplementary Materials). It can be seen that the *E*_ZPE_ values of the SACs with hydrogen atoms adsorbed on the top of the C atoms (around 0.24 eV) are larger than those of the SACs adsorbing H on the top of the V atoms (0.17–0.19 eV) or imported transition-metal atoms (no more than 0.20 eV). In addition, the *TS*_H_ values are smaller than 0.1 eV, indicating less influence on the free energy compared with *E*_ZPE_.

The Δ*G*_H*_ values of TM@(4 × 4)-V_3_C_3_-V_surf-C_ at different adsorption sites are listed in Table 5. For each TM@(4 × 4)-V_3_C_3_-V_surf-C_, the Δ*G*_H*_ values of the active sites on the top of the C atoms are lower than those of the V^2^ and V^3^ sites. In addition, adsorption sites V^2^ and V^3^ exhibit similar values of Gibbs free energy. Apart from Mn@(4 × 4)-V_3_C_3_-V_surf-C_, other TM@(4 × 4)-V_3_C_3_-V_surf-C_ possess active sites with a |Δ*G*_H*_| smaller than 0.2 eV, suggesting their great HER catalytic ability. Specifically, (Ti and Co)@(4 × 4)-V_3_C_3_-V_surf-C_ display excellent catalytic performance at sites C^1^, C^2^, C^3^, C^4^, C^5^, and TM^1^. Ni@(4 × 4)-V_3_C_3_-V_surf-C_ also has six sites (C^1^, C^3^, C^4^, C^5^, V^1^, and TM^1^) exhibiting excellent catalytic performance. (V, Fe and Cu)@(4 × 4)-V_3_C_3_-V_surf-C_ have more than two active sites with a |Δ*G*_H*_| smaller than 0.2 eV. Only Cr@(4 × 4)-V_3_C_3_-V_surf-C_ has one adsorption site with great catalytic performance. These results indicate that most of the designed TM@(4 × 4)-V_3_C_3_-V_surf-C_ have potential as promising HER catalysts.

The Δ*G*_H*_ values of TM@(4 × 4)-V_2_C_2_-V_surf-V_ suggest that they are all outstanding HER catalysts (Table 6). All the adsorption sites on the top of the C atoms exhibit a |Δ*G*_H*_| smaller than 0.2 eV for all the TM@(4 × 4)-V_2_C_2_-V_surf-V_ except Co@(4 × 4)-V_2_C_2_-V_surf-V_. Co@(4 × 4)-V_2_C_2_-V_surf-V_ shows excellent HER catalytic performance at sites C^2^ and C^3^. Different from the sites on the top of the C atoms, the sites on the top of the V atoms and anchored transition-metal atoms have values not less than 0.45 eV and display inefficient catalytic activity. On the whole, we can conclude that all the TM@(4 × 4)-V_2_C_2_-V_surf-V_ can act as great HER catalysts.

Overall, V_3_C_3_-based SACs have better HER catalytic capacity than their V_2_C_2_-based counterparts. Based on the |Δ*G*_H*_| values, apart from Mn@(4 × 4)-V_3_C_3_-V_surf-C_, the HER catalytic abilities of other V_3_C_3_-based SACs are comparable to those of Pt- and Ru-based catalysts [51,52], suggesting the potential to replace precious-metal-based catalysts for HER. The |Δ*G*_H*_| value of every adsorption site is lower than that of MoS_2_ (2.0 eV) [50]. In addition, the minimum |Δ*G*_H*_| values of most V_3_C_3_-based SACs are comparable to the Δ*G*_H*_ of Cr_2_TiC_2_O_2_ and Cr_2_VC_2_O_2_ MXenes [53]. Cr_2_TiC_2_O_2_ possesses excellent HER catalytic performance when hydrogen coverage is 1/4 (Δ*G*_H*_ = −0.17 eV), 3/8 (Δ*G*_H*_ = 0.16 eV), and 1/2 ML (Δ*G*_H*_ = 0.20 eV), while Cr_2_VC_2_O_2_ exhibits great HER catalytic ability for 3/8 hydrogen coverage (Δ*G*_H*_ = −0.03 eV). This indicates that transition-metal carbides have potential application value in HER catalysis.

## 4. Summary

In summary, we have constructed a battery of HER SACs by importing transition-metal atoms into the carbon and vanadium vacancies of 2D tetragonal V_2_C_2_ and V_3_C_3_. By means of first-principles computations, the feasibility of applying these SACs in HER catalysis was examined. The results indicated that all the SACs are thermally stable and conductive, which is favorable to catalysis. The Gibbs free energy change during hydrogen adsorption was used to evaluate their catalytic ability. Among the V_2_C_2_-based SACs, Mn@(4 × 4)-V_2_C_2_-V_C_ possesses eight adsorption sites with excellent HER catalytic ability, and (V, Cr, Fe, Ni and Cu)@(4 × 4)-V_2_C_2_-V_C_ also exhibit great HER catalytic performance at some active sites. These excellent V_2_C_2_-based SACs have a smaller |Δ*G*_H*_| at some sites than pristine V_2_C_2_, Mo_2_B_2_, and MoS_2_, indicating the great advantage of SACs. Among the V_3_C_3_-based SACs, apart from Mn@(4 × 4)-V_3_C_3_-V_surf-C_, all the SACs can act as outstanding HER catalysts. The minimum |Δ*G*_H*_| values of these outstanding V_3_C_3_-based SACs are comparable to the Δ*G*_H*_ of Cr_2_TiC_2_O_2_ and Cr_2_VC_2_O_2_ MXenes. Overall, these great V_2_C_2_- and V_3_C_3_-based SACs can be compared with the best-known Pt-based HER catalysts. It should be noted that this is a pure theoretical study as the V_2_C_2_ and V_3_C_3_ slabs have not been successfully synthesized in the laboratory. This work offers researchers an incentive to synthesize these two materials. After successfully synthesizing these two materials, the stability of them in real electrochemical HER environments should be ensured before practical application.

## Figures and Tables

**Table 1:** The binding energies *E*_bind_ (eV) of transition-metal-atom-substituted C or V monovacancies of (4 × 4)-V_2_C_2_.

	Ti	V	Cr	Mn	Fe	Co	Ni	Cu
C monovacancy	1.62	2.22	2.27	2.12	1.51	1.58	1.11	0.92
V monovacancy	−5.02		−3.81	−3.34	−2.84	−2.49	−2.14	−1.14

**Table 2:** The binding energies *E*_bind_ (eV) of transition-metal-atom-substituted C_surf_ or V_surf_ monovacancies of (4 × 4)-V_3_C_3_.

	Ti	V	Cr	Mn	Fe	Co	Ni	Cu
C_surf_ monovacancy	1.72	2.62	2.90	2.78	2.41	1.86	1.04	0.77
V_surf_ monovacancy	−5.61		−3.90	−3.38	−2.80	−2.31	−2.03	−1.17

**Table 3:** Calculated Gibbs free energies at different adsorption sites for TM@(4 × 4)-V_2_C_2_-V_C_. The units are in electron volts.

	C^1^	C^2^	C^3^	C^4^	C^5^	V^1^	V^2^	V^3^	TM^1^
Ti@(4 × 4)-V_2_C_2_-V_C_	0.38	0.47	0.53	0.45	0.42	−0.33	0.52	0.53	0.30
V@(4 × 4)-V_2_C_2_-V_C_	0.43	0.45	0.51	0.47	0.43	−0.23	0.52	0.53	0.13
Cr@(4 × 4)-V_2_C_2_-V_C_	0.45	0.48	0.45	0.48	0.47	−0.22	0.53	0.53	−0.06
Mn@(4 × 4)-V_2_C_2_-V_C_	−0.13	0.00	0.02	−0.04	−0.05	−0.78	0.07	0.06	−0.04
Fe@(4 × 4)-V_2_C_2_-V_C_	0.40	0.49	0.49	0.45	0.43	−0.14	0.54	0.53	0.04
Co@(4 × 4)-V_2_C_2_-V_C_	0.35	0.43	0.43	0.41	0.40	−0.23	0.50	0.48	−0.23
Ni@(4 × 4)-V_2_C_2_-V_C_	0.39	0.47	0.48	0.46	0.41	−0.18	0.56	0.52	−0.19
Cu@(4 × 4)-V_2_C_2_-V_C_	0.35	0.51	0.49	0.42	0.42	−0.04	0.53	0.51	0.19

**Table 4:** Calculated Gibbs free energies at different adsorption sites for TM@(4 × 4)-V_2_C_2_-V_V_. The units are in electron volts.

	C^1^	C^2^	C^3^	V^1^	V^2^	V^3^	V^4^	V^5^	TM^1^
Ti@(4 × 4)-V_2_C_2_-V_V_	0.43	0.46	0.47	0.52	0.54	0.52	0.52	0.51	0.78
Cr@(4 × 4)-V_2_C_2_-V_V_	0.46	0.46	0.47	0.51	0.50	0.52	0.50	0.51	0.31
Mn@(4 × 4)-V_2_C_2_-V_V_	0.42	0.45	0.47	0.57	0.50	0.51	0.51	0.53	0.31
Fe@(4 × 4)-V_2_C_2_-V_V_	0.38	0.46	0.51	0.59	0.50	0.53	0.50	0.54	0.35
Co@(4 × 4)-V_2_C_2_-V_V_	0.33	0.51	0.55	0.67	0.54	0.58	0.56	0.60	0.47
Ni@(4 × 4)-V_2_C_2_-V_V_	0.34	0.46	0.53	0.67	0.51	0.57	0.52	0.57	0.91
Cu@(4 × 4)-V_2_C_2_-V_V_	0.44	0.47	0.51	0.63	0.50	0.62	0.52	0.56	1.48

**Table 5:** Calculated Gibbs free energies at different adsorption sites for TM@(4 × 4)-V_3_C_3_-V_surf-C_. The units are in electron volts.

	C^1^	C^2^	C^3^	C^4^	C^5^	V^1^	V^2^	V^3^	TM^1^
Ti@(4 × 4)-V_3_C_3_-V_surf-C_	−0.16	0.19	0.17	0.09	0.02	−0.21	0.66	0.66	0.18
V@(4 × 4)-V_3_C_3_-V_surf-C_	−0.36	0.13	0.17	0.09	0.03	−0.21	0.66	0.66	0.05
Cr@(4 × 4)-V_3_C_3_-V_surf-C_	−0.91	−0.70	−0.77	−0.87	−0.88	−1.14	−0.30	−0.32	−0.09
Mn@(4 × 4)-V_3_C_3_-V_surf-C_	−1.23	−1.04	−1.10	−1.18	−1.20	−1.46	−0.62	−0.63	−1.07
Fe@(4 × 4)-V_3_C_3_-V_surf-C_	−0.71	−0.63	−0.64	−0.73	−0.75	−0.89	−0.14	−0.16	−0.01
Co@(4 × 4)-V_3_C_3_-V_surf-C_	−0.14	0.02	0.02	−0.06	0.06	−0.23	0.51	0.49	−0.08
Ni@(4 × 4)-V_3_C_3_-V_surf-C_	0.15	0.21	0.19	0.10	0.09	−0.03	0.68	0.66	0.10
Cu@(4 × 4)-V_3_C_3_-V_surf-C_	0.23	0.28	0.21	0.12	0.11	0.04	0.68	0.66	0.41

**Table 6:** Calculated Gibbs free energies at different adsorption sites for TM@(4 × 4)-V_3_C_3_-V_surf-V_. The units are in electron volts.

	C^1^	C^2^	C^3^	V^1^	V^2^	V^3^	V^4^	V^5^	TM^1^
Ti@(4 × 4)-V_3_C_3_-V_surf-V_	0.15	0.12	0.07	0.63	0.68	0.63	0.63	0.63	0.94
Cr@(4 × 4)-V_3_C_3_-V_surf-V_	−0.04	0.04	0.08	0.65	0.61	0.64	0.63	0.63	0.45
Mn@(4 × 4)-V_3_C_3_-V_surf-V_	−0.05	0.08	0.10	0.65	0.65	0.65	0.64	0.63	0.57
Fe@(4 × 4)-V_3_C_3_-V_surf-V_	−0.12	0.08	0.12	0.66	0.65	0.65	0.64	0.63	0.59
Co@(4 × 4)-V_3_C_3_-V_surf-V_	−0.25	0.03	0.08	0.64	0.61	0.66	0.61	0.63	0.60
Ni@(4 × 4)-V_3_C_3_-V_surf-V_	−0.18	0.08	0.13	0.66	0.65	0.67	0.64	0.62	0.98
Cu@(4 × 4)-V_3_C_3_-V_surf-V_	−0.04	0.10	0.10	0.64	0.67	0.67	0.65	0.63	1.37

## Data Availability

The original contributions presented in the study are included in the article/supplementary materials. Further inquiries can be directed to the corresponding authors.

## Supplementary Materials

The following supporting information can be downloaded at https://www.mdpi.com/article/10.3390/ma18050931/s1. Figure S1: Top and side views of the optimized configurations of (a) Ti@(4 × 4)-V_2_C_2_-V_C_, (b) V@(4 × 4)-V_2_C_2_-V_C_, (c) Cr@(4 × 4)-V_2_C_2_-V_C_, (d) Mn@(4 × 4)-V_2_C_2_-V_C_, (e) Fe@(4 × 4)-V_2_C_2_-V_C_, (f) Co@(4 × 4)-V_2_C_2_-V_C_, (g) Ni@(4 × 4)-V_2_C_2_-V_C_ and (h) Cu@(4 × 4)-V_2_C_2_-V_C_; Figure S2: Top and side views of the optimized configurations of (a) Ti@(4 × 4)-V_2_C_2_-V_V_, (b) Cr@(4 × 4)-V_2_C_2_-V_V_, (c) Mn@(4 × 4)-V_2_C_2_-V_V_, (d) Fe@(4 × 4)-V_2_C_2_-V_V_, (e) Co@(4 × 4)-V_2_C_2_-V_V_, (f) Ni@(4 × 4)-V_2_C_2_-V_V_ and (g) Cu@(4 × 4)-V_2_C_2_-V_V_; Figure S3: Top and side views of the optimized configurations of (a) Ti@(4 × 4)-V_3_C_3_-V_surf-C_, (b) V@(4 × 4)-V_3_C_3_-V_surf-C_, (c) Cr@(4 × 4)-V_3_C_3_-V_surf-C_, (d) Mn@(4 × 4)-V_3_C_3_-V_surf-C_, (e) Fe@(4 × 4)-V_3_C_3_-V_surf-C_, (f) Co@(4 × 4)-V_3_C_3_-V_surf-C_, (g) Ni@(4 × 4)-V_3_C_3_-V_surf-C_ and (h) Cu@(4 × 4)-V_3_C_3_-V_surf-C_; Figure S4: Top and side views of the optimized configurations of (a) Ti@(4 × 4)-V_3_C_3_-V_surf-V_, (b) Cr@(4 × 4)-V_3_C_3_-V_surf-V_, (c) Mn@(4 × 4)-V_3_C_3_-V_surf-V_, (d) Fe@(4 × 4)-V_3_C_3_-V_surf-V_, (e) Co@(4 × 4)-V_3_C_3_-V_surf-V_, (f) Ni@(4 × 4)-V_3_C_3_-V_surf-V_ and (g) Cu@(4 × 4)-V_3_C_3_-V_surf-V_; Figure S5: Energies as a function of time of (a) Ti@(4 × 4)-V_2_C_2_-V_C_, (b) V@(4 × 4)-V_2_C_2_-V_C_, (c) Cr@(4 × 4)-V_2_C_2_-V_C_, (d) Mn@(4 × 4)-V_2_C_2_-V_C_, (e) Fe@(4 × 4)-V_2_C_2_-V_C_, (f) Co@(4 × 4)-V_2_C_2_-V_C_, (g) Ni@(4 × 4)-V_2_C_2_-V_C_ and (h) Cu@(4 × 4)-V_2_C_2_-V_C_. during the AIMD simulations (inset: the configurations after 10 ps AIMD simulations); Figure S6: Energies as a function of time of (a) Ti@(4 × 4)-V_2_C_2_-V_V_, (b) Cr@(4 × 4)-V_2_C_2_-V_V_, (c) Mn@(4 × 4)-V_2_C_2_-V_V_, (d) Fe@(4 × 4)-V_2_C_2_-V_V_, (e) Co@(4 × 4)-V_2_C_2_-V_V_, (f) Ni@(4 × 4)-V_2_C_2_-V_V_ and (g) Cu@(4 × 4)-V_2_C_2_-V_V_. during the AIMD simulations (inset: the configurations after 10 ps AIMD simulations); Figure S7: Energies as a function of time of (a) Ti@(4 × 4)-V_3_C_3_-V_surf-C_, (b) V@(4 × 4)-V_3_C_3_-V_surf-C_, (c) Cr@(4 × 4)-V_3_C_3_-V_surf-C_, (d) Mn@(4 × 4)-V_3_C_3_-V_surf-C_, (e) Fe@(4 × 4)-V_3_C_3_-V_surf-C_, (f) Co@(4 × 4)-V_3_C_3_-V_surf-C_, (g) Ni@(4 × 4)-V_3_C_3_-V_surf-C_ and (h) Cu@(4 × 4)-V_3_C_3_-V_surf-C_. during the AIMD simulations (inset: the configurations after 10 ps AIMD simulations); Figure S8: Energies as a function of time of (a) Ti@(4 × 4)-V_3_C_3_-V_surf-V_, (b) Cr@(4 × 4)-V_3_C_3_-V_surf-V_, (c) Mn@(4 × 4)-V_3_C_3_-V_surf-V_, (d) Fe@(4 × 4)-V_3_C_3_-V_surf-V_, (e) Co@(4 × 4)-V_3_C_3_-V_surf-V_, (f) Ni@(4 × 4)-V_3_C_3_-V_surf-V_ and (g) Cu@(4 × 4)-V_3_C_3_-V_surf-V_. during the AIMD simulations (inset: the configurations after 10 ps AIMD simulations); Figure S9: Total and partial density of states of (a) (4 × 4)-V_2_C_2_-V_C_, (b) Ti@(4 × 4)-V_2_C_2_-V_C_, (c) V@(4 × 4)-V_2_C_2_-V_C_, (d) Cr@(4 × 4)-V_2_C_2_-V_C_, (e) Mn@(4 × 4)-V_2_C_2_-V_C_, (f) Fe@(4 × 4)-V_2_C_2_-V_C_, (g) Co@(4 × 4)-V_2_C_2_-V_C_, (h) Ni@(4 × 4)-V_2_C_2_-V_C_ and (i) Cu@(4 × 4)-V_2_C_2_-V_C_. The Fermi level is set to zero and marked with the dashed line; Figure S10: Total and partial density of states of (a) (4 × 4)-V_2_C_2_-V_V_, (b) Ti@(4 × 4)-V_2_C_2_-V_V_, (c) Cr@(4 × 4)-V_2_C_2_-V_V_, (d) Mn@(4 × 4)-V_2_C_2_-V_V_, (e) Fe@(4 × 4)-V_2_C_2_-V_V_, (f) Co@(4 × 4)-V_2_C_2_-V_V_, (g) Ni@(4 × 4)-V_2_C_2_-V_V_ and (h) Cu@(4 × 4)-V_2_C_2_-V_V_. The Fermi level is set to zero and marked with the dashed line; Figure S11: Total and partial density of states of (a) (4 × 4)-V_2_C_2_-V_surf-C_, (b) Ti@(4 × 4)-V_2_C_2_-V_surf-C_, (c) V@(4 × 4)-V_2_C_2_-V_surf-C_, (d) Cr@(4 × 4)-V_2_C_2_-V_surf-C_, (e) Mn@(4 × 4)-V_2_C_2_-V_surf-C_, (f) Fe@(4 × 4)-V_2_C_2_-V_surf-C_, (g) Co@(4 × 4)-V_2_C_2_-V_surf-C_, (h) Ni@(4 × 4)-V_2_C_2_-V_surf-C_ and (i) Cu@(4 × 4)-V_2_C_2_-V_surf-C_. The Fermi level is set to zero and marked with the dashed line; Figure S12: Total and partial density of states of (a) (4 × 4)-V_2_C_2_-V_surf-V_, (b) Ti@(4 × 4)-V_2_C_2_-V_surf-V_, (c) Cr@(4 × 4)-V_2_C_2_-V_surf-V_, (d) Mn@(4 × 4)-V_2_C_2_-V_surf-V_, (e) Fe@(4 × 4)-V_2_C_2_-V_surf-V_, (f) Co@(4 × 4)-V_2_C_2_-V_surf-V_, (g) Ni@(4 × 4)-V_2_C_2_-V_surf-V_ and (h) Cu@(4 × 4)-V_2_C_2_-V_surf-V_. The Fermi level is set to zero and marked with the dashed line; Table S1: The lattice constants (Å) of TM@(4 × 4)-V_2_C_2_-V_C_; Table S2: The lattice constants (Å) of TM@(4 × 4)-V_2_C_2_-V_V_; Table S3: The lattice constants (Å) of TM@(4 × 4)-V_3_C_3_-V_surf-C_; Table S4: The lattice constants (Å) of TM@(4 × 4)-V_3_C_3_-V_surf-V_; Table S5: Calculated energies (*E*), zero-point energies (*E*zpe), vibrational entropy (*TS*_H_), Gibbs free energies (*G*) and Gibbs free energy differences (Δ*G*) of Ti@(4 × 4)-V_2_C_2_-V_C_ at different adsorption sites; Table S6: Calculated energies (*E*), zero-point energies (*E*zpe), vibrational entropy (*TS*_H_), Gibbs free energies (*G*) and Gibbs free energy differences (Δ*G*) of V@(4 × 4)-V_2_C_2_-V_C_ at different adsorption sites; Table S7: Calculated energies (*E*), zero-point energies (*E*zpe), vibrational entropy (*TS*_H_), Gibbs free energies (*G*) and Gibbs free energy differences (Δ*G*) of Cr@(4 × 4)-V_2_C_2_-V_C_ at different adsorption sites; Table S8: Calculated energies (*E*), zero-point energies (*E*zpe), vibrational entropy (*TS*_H_), Gibbs free energies (*G*) and Gibbs free energy differences (Δ*G*) of Mn@(4 × 4)-V_2_C_2_-V_C_ at different adsorption sites; Table S9: Calculated energies (*E*), zero-point energies (*E*zpe), vibrational entropy (*TS*_H_), Gibbs free energies (*G*) and Gibbs free energy differences (Δ*G*) of Fe@(4 × 4)-V_2_C_2_-V_C_ at different adsorption sites; Table S10: Calculated energies (*E*), zero-point energies (*E*zpe), vibrational entropy (*TS*_H_), Gibbs free energies (*G*) and Gibbs free energy differences (Δ*G*) of Co@(4 × 4)-V_2_C_2_-V_C_ at different adsorption sites; Table S11: Calculated energies (*E*), zero-point energies (*E*zpe), vibrational entropy (*TS*_H_), Gibbs free energies (*G*) and Gibbs free energy differences (Δ*G*) of Ni@(4 × 4)-V_2_C_2_-V_C_ at different adsorption sites; Table S12: Calculated energies (*E*), zero-point energies (*E*zpe), vibrational entropy (*TS*_H_), Gibbs free energies (*G*) and Gibbs free energy differences (Δ*G*) of Cu@(4 × 4)-V_2_C_2_-V_C_ at different adsorption sites; Table S13: Calculated energies (*E*), zero-point energies (*E*zpe), vibrational entropy (*TS*_H_), Gibbs free energies (*G*) and Gibbs free energy differences (Δ*G*) of Ti@(4 × 4)-V_2_C_2_-V_V_ at different adsorption sites; Table S14: Calculated energies (*E*), zero-point energies (*E*zpe), vibrational entropy (*TS*_H_), Gibbs free energies (*G*) and Gibbs free energy differences (Δ*G*) of Cr@(4 × 4)-V_2_C_2_-V_V_ at different adsorption sites; Table S15: Calculated energies (*E*), zero-point energies (*E*zpe), vibrational entropy (*TS*_H_), Gibbs free energies (*G*) and Gibbs free energy differences (Δ*G*) of Mn@(4 × 4)-V_2_C_2_-V_V_ at different adsorption sites; Table S16: Calculated energies (*E*), zero-point energies (*E*zpe), vibrational entropy (*TS*_H_), Gibbs free energies (*G*) and Gibbs free energy differences (Δ*G*) of Fe@(4 × 4)-V_2_C_2_-V_V_ at different adsorption sites; Table S17: Calculated energies (*E*), zero-point energies (*E*zpe), vibrational entropy (*TS*_H_), Gibbs free energies (*G*) and Gibbs free energy differences (Δ*G*) of Co@(4 × 4)-V_2_C_2_-V_V_ at different adsorption sites; Table S18: Calculated energies (*E*), zero-point energies (*E*zpe), vibrational entropy (*TS*_H_), Gibbs free energies (*G*) and Gibbs free energy differences (Δ*G*) of Ni@(4 × 4)-V_2_C_2_-V_V_ at different adsorption sites; Table S19: Calculated energies (*E*), zero-point energies (*E*zpe), vibrational entropy (*TS*_H_), Gibbs free energies (*G*) and Gibbs free energy differences (Δ*G*) of Cu@(4 × 4)-V_2_C_2_-V_V_ at different adsorption sites; Table S20: Calculated energies (*E*), zero-point energies (*E*zpe), vibrational entropy (*TS*_H_), Gibbs free energies (*G*) and Gibbs free energy differences (Δ*G*) of Ti@(4 × 4)-V_3_C_3_-V_surf-C_ at different adsorption sites; Table S21: Calculated energies (*E*), zero-point energies (*E*zpe), vibrational entropy (*TS*_H_), Gibbs free energies (*G*) and Gibbs free energy differences (Δ*G*) of V@(4 × 4)-V_3_C_3_-V_surf-C_ at different adsorption sites; Table S22: Calculated energies (*E*), zero-point energies (*E*zpe), vibrational entropy (*TS*_H_), Gibbs free energies (*G*) and Gibbs free energy differences (Δ*G*) of Cr@(4 × 4)-V_3_C_3_-V_surf-C_ at different adsorption sites; Table S23: Calculated energies (*E*), zero-point energies (*E*zpe), vibrational entropy (*TS*_H_), Gibbs free energies (*G*) and Gibbs free energy differences (Δ*G*) of Mn@(4 × 4)-V_3_C_3_-V_surf-C_ at different adsorption sites; Table S24: Calculated energies (*E*), zero-point energies (*E*zpe), vibrational entropy (*TS*_H_), Gibbs free energies (*G*) and Gibbs free energy differences (Δ*G*) of Fe@(4 × 4)-V_3_C_3_-V_surf-C_ at different adsorption sites; Table S25: Calculated energies (*E*), zero-point energies (*E*zpe), vibrational entropy (*TS*_H_), Gibbs free energies (*G*) and Gibbs free energy differences (Δ*G*) of Co@(4 × 4)-V_3_C_3_-V_surf-C_ at different adsorption sites; Table S26: Calculated energies (*E*), zero-point energies (*E*zpe), vibrational entropy (*TS*_H_), Gibbs free energies (*G*) and Gibbs free energy differences (Δ*G*) of Ni@(4 × 4)-V_3_C_3_-V_surf-C_ at different adsorption sites; Table S27: Calculated energies (*E*), zero-point energies (*E*zpe), vibrational entropy (*TS*_H_), Gibbs free energies (*G*) and Gibbs free energy differences (Δ*G*) of Cu@(4 × 4)-V_3_C_3_-V_surf-C_ at different adsorption sites; Table S28: Calculated energies (*E*), zero-point energies (*E*zpe), vibrational entropy (*TS*_H_), Gibbs free energies (*G*) and Gibbs free energy differences (Δ*G*) of Ti@(4 × 4)-V_3_C_3_-V_surf-V_ at different adsorption sites; Table S29: Calculated energies (*E*), zero-point energies (*E*zpe), vibrational entropy (*TS*_H_), Gibbs free energies (*G*) and Gibbs free energy differences (Δ*G*) of Cr@(4 × 4)-V_3_C_3_-V_surf-V_ at different adsorption sites; Table S30: Calculated energies (*E*), zero-point energies (*E*zpe), vibrational entropy (*TS*_H_), Gibbs free energies (*G*) and Gibbs free energy differences (Δ*G*) of Mn@(4 × 4)-V_3_C_3_-V_surf-V_ at different adsorption sites; Table S31: Calculated energies (*E*), zero-point energies (*E*zpe), vibrational entropy (*TS*_H_), Gibbs free energies (*G*) and Gibbs free energy differences (Δ*G*) of Fe@(4 × 4)-V_3_C_3_-V_surf-V_ at different adsorption sites; Table S32: Calculated energies (*E*), zero-point energies (*E*zpe), vibrational entropy (*TS*_H_), Gibbs free energies (*G*) and Gibbs free energy differences (Δ*G*) of Co@(4 × 4)-V_3_C_3_-V_surf-V_ at different adsorption sites; Table S33: Calculated energies (*E*), zero-point energies (*E*zpe), vibrational entropy (*TS*_H_), Gibbs free energies (*G*) and Gibbs free energy differences (Δ*G*) of Ni@(4 × 4)-V_3_C_3_-V_surf-V_ at different adsorption sites; Table S34: Calculated energies (*E*), zero-point energies (*E*zpe), vibrational entropy (*TS*_H_), Gibbs free energies (*G*) and Gibbs free energy differences (Δ*G*) of Cu@(4 × 4)-V_3_C_3_-V_surf-V_ at different adsorption sites.
