# Supplementary material for: Theoretical Investigation of Single-Atom Catalysts for Hydrogen Evolution Reaction Based on Two-Dimensional Tetragonal V2C2 and V3C3
Source: Materials (Basel). 2025 Feb 20;18(5):931. doi: 10.3390/ma18050931 (PMC11901156; doi:10.3390/ma18050931)
Supplement: Supplementary file 1 [file materials-18-00931-s001.zip › materials-3476229-supplementary.pdf]

Electronic Supplementary Information

Theoretical Investigation of Single-Atom Catalysts for Hydrogen Evolution Reaction Based on Two-Dimensional Tetragonal V_2C_2 and V_3C_3

Bo Xue [1,*], Qingfeng Zeng [2,3,4], Shuyin Yu [2,3] and Kehe Su [5,*]

1 School of Physical Science and Technology, Northwestern Polytechnical University, Xi'an 710129, China
2 MSEA International Institute for Materials Genome, Langfang 065500, China; zengqf@dianyunkeji.com (Q.Z.); yusy@dianyunkeji.com (S.Y.)
3 Particle Cloud Biotechnology (Hangzhou) Co., Ltd., Hangzhou 310018, China
4 Science and Technology on Thermostructural Composite Materials Laboratory, Northwestern Polytechnical University, Xi'an 710072, China
5 School of Chemistry and Chemical Engineering, Northwestern Polytechnical University, Xi'an 710129, China
* Correspondence: xuebo@mail.nwpu.edu.cn (B.X.); sukehe@nwpu.edu.cn (K.S.)

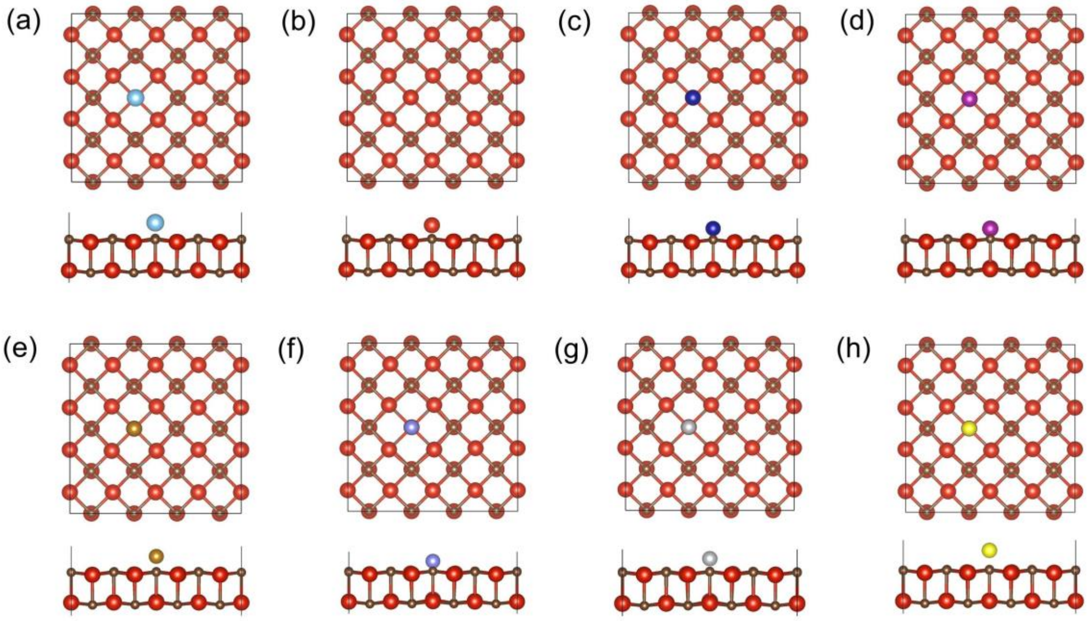

Figure S1. Top and side views of the optimized configurations of (**a**) Ti@(4 × 4)-V_2C_2-V_C, (**b**) V@(4 × 4)-V_2C_2-V_C, (**c**) Cr@(4 × 4)-V_2C_2-V_C, (**d**) Mn@(4 × 4)-V_2C_2-V_C, (**e**) Fe@(4 × 4)-V_2C_2-V_C, (**f**) Co@(4 × 4)-V_2C_2-V_C, (**g**) Ni@(4 × 4)-V_2C_2-V_C and (**h**) Cu@(4 × 4)-V_2C_2-V_C.

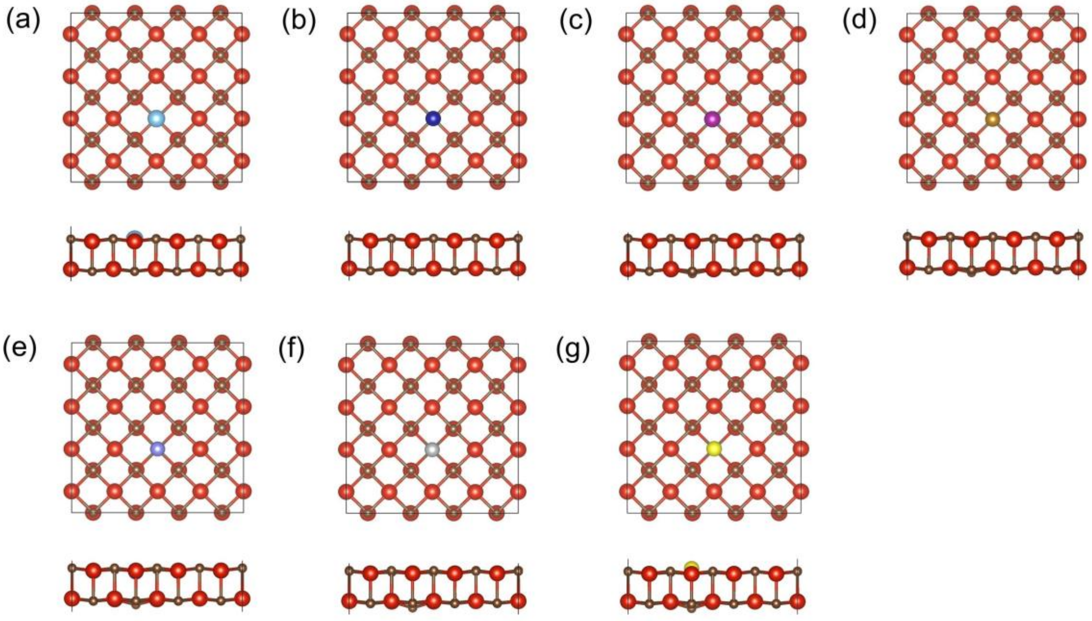

Figure S2. Top and side views of the optimized configurations of (**a**) Ti@(4 × 4)-V_2C_2-V_V, (**b**) Cr@(4 × 4)-V_2C_2-V_V, (**c**) Mn@(4 × 4)-V_2C_2-V_V, (**d**) Fe@(4 × 4)-V_2C_2-V_V, (**e**) Co@(4 × 4)-V_2C_2-V_V, (**f**) Ni@(4 × 4)-V_2C_2-V_V and (**g**) Cu@(4 × 4)-V_2C_2-V_V.

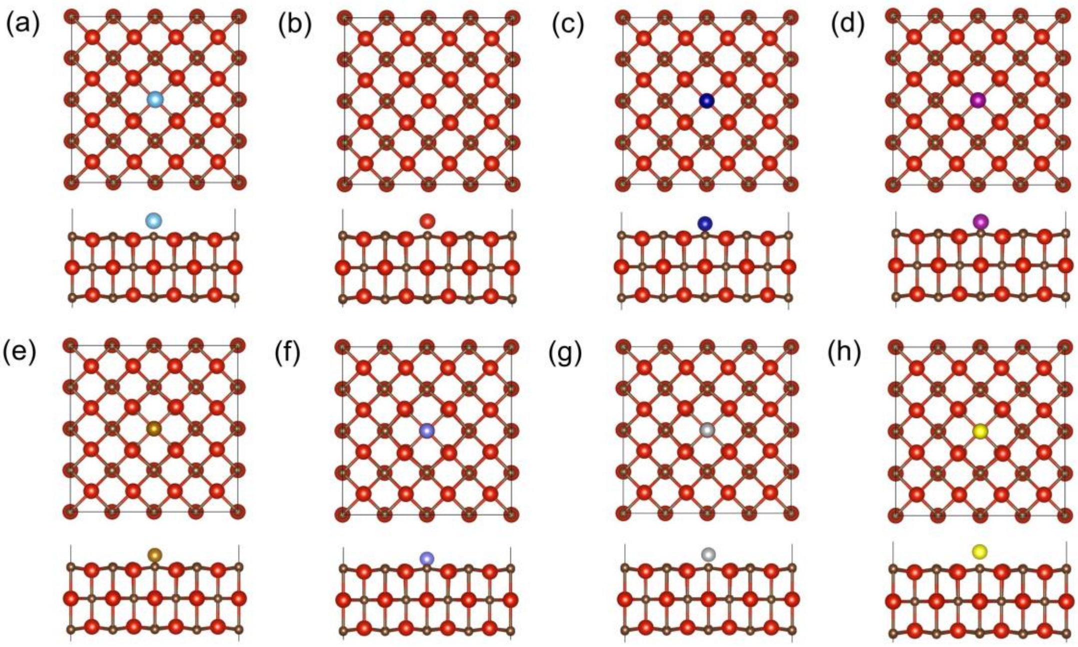

Figure S3. Top and side views of the optimized configurations of (**a**) Ti@(4 × 4)-V_3C_3-$V_{surf\text{-}C}$, (**b**) V@(4 × 4)-V_3C_3-$V_{surf\text{-}C}$, (**c**) Cr@(4 × 4)-V_3C_3-$V_{surf\text{-}C}$, (**d**) Mn@(4 × 4)-V_3C_3-$V_{surf\text{-}C}$, (**e**) Fe@(4 × 4)-V_3C_3-$V_{surf\text{-}C}$, (**f**) Co@(4 × 4)-V_3C_3-$V_{surf\text{-}C}$, (**g**) Ni@(4 × 4)-V_3C_3-$V_{surf\text{-}C}$ and (**h**) Cu@(4 × 4)-V_3C_3-$V_{surf\text{-}C}$.

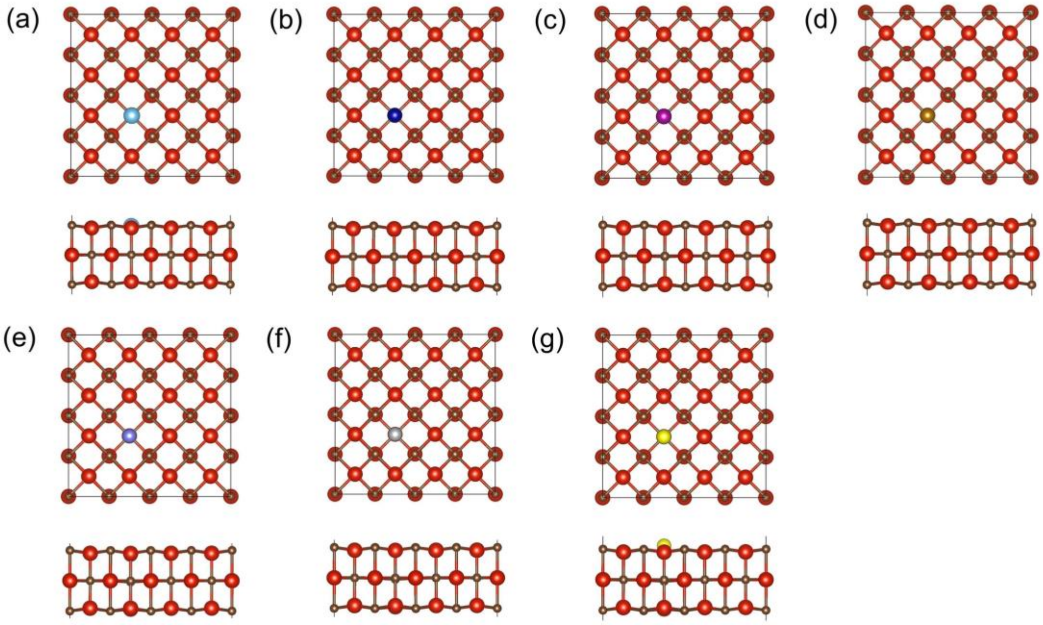

Figure S4. Top and side views of the optimized configurations of (**a**) Ti@(4 × 4)-V_3C_3-$V_{surf\text{-}V}$, (**b**) Cr@(4 × 4)-V_3C_3-$V_{surf\text{-}V}$, (**c**) Mn@(4 × 4)-V_3C_3-$V_{surf\text{-}V}$, (**d**) Fe@(4 × 4)-V_3C_3-$V_{surf\text{-}V}$, (**e**) Co@(4 × 4)-V_3C_3-$V_{surf\text{-}V}$, (**f**) Ni@(4 × 4)-V_3C_3-$V_{surf\text{-}V}$ and (**g**) Cu@(4 × 4)-V_3C_3-$V_{surf\text{-}V}$.

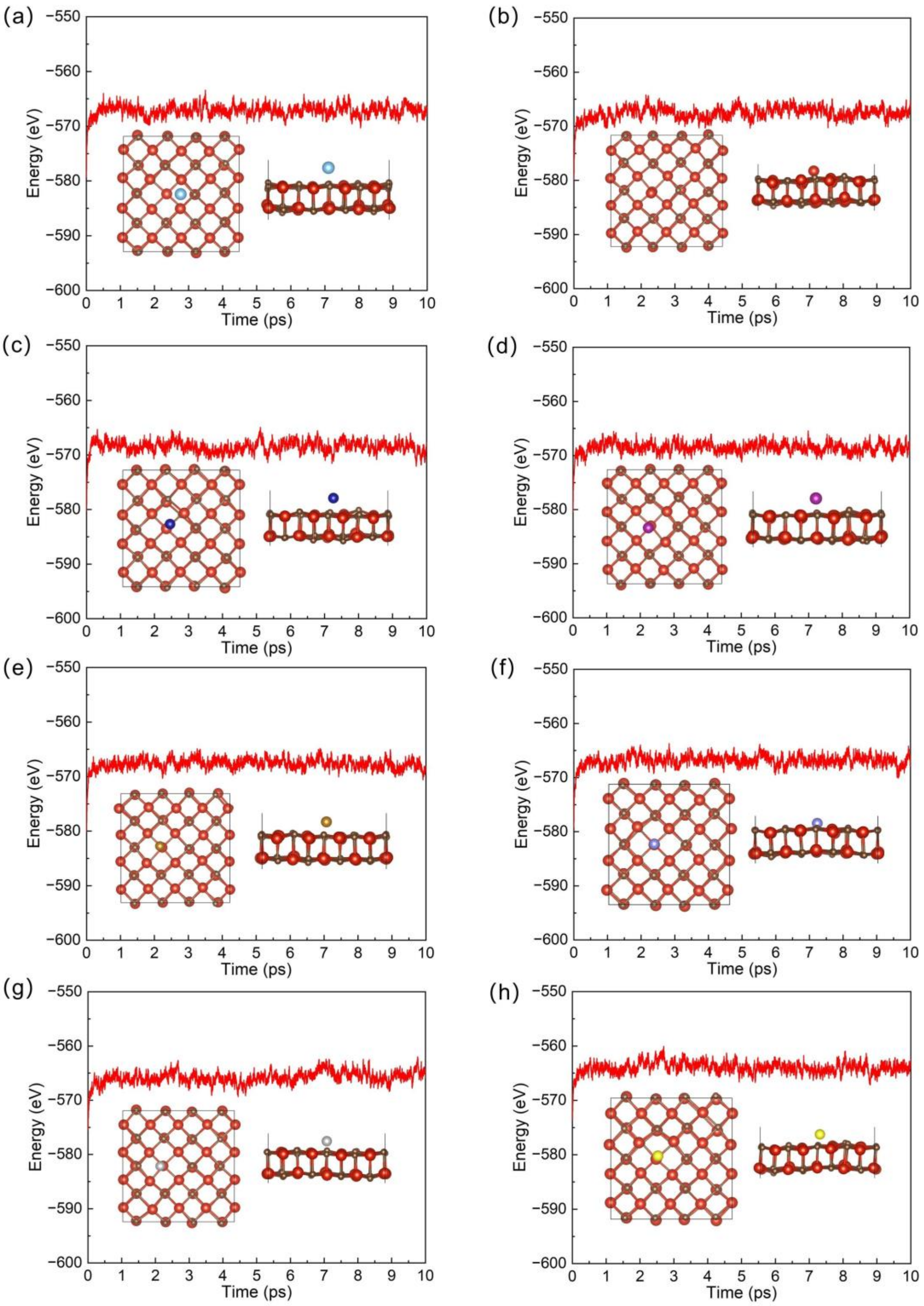

Figure S5. Energies as a function of time of (**a**) Ti@(4 × 4)-V_2C_2-V_C, (**b**) V@(4 × 4)-V_2C_2-V_C, (**c**) Cr@(4 × 4)-V_2C_2-V_C, (**d**) Mn@(4 × 4)-V_2C_2-V_C, (**e**) Fe@(4 × 4)-V_2C_2-V_C, (**f**) Co@(4 × 4)-V_2C_2-V_C, (**g**) Ni@(4 × 4)-V_2C_2-V_C and (**h**) Cu@(4 × 4)-V_2C_2-V_C. during the AIMD simulations (inset: the configurations after 10 ps AIMD simulations).

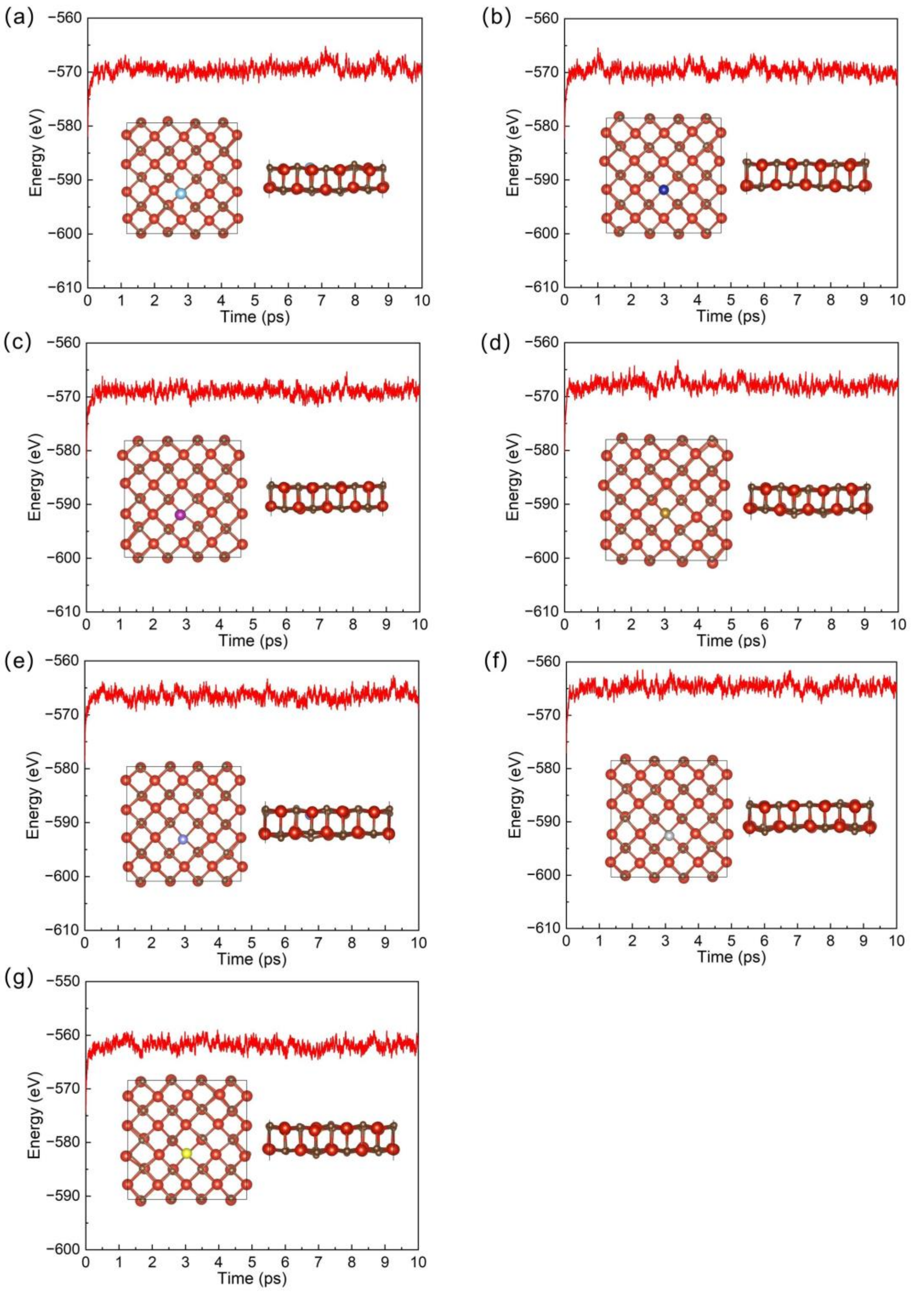

Figure S6. Energies as a function of time of (**a**) Ti@(4 × 4)-V_2C_2-V_V, (**b**) Cr@(4 × 4)-V_2C_2-V_V, (**c**) Mn@(4 × 4)-V_2C_2-V_V, (**d**) Fe@(4 × 4)-V_2C_2-V_V, (**e**) Co@(4 × 4)-V_2C_2-V_V, (**f**) Ni@(4 × 4)-V_2C_2-V_V and (**g**) Cu@(4 × 4)-V_2C_2-V_V. during the AIMD simulations (inset: the configurations after 10 ps AIMD simulations).

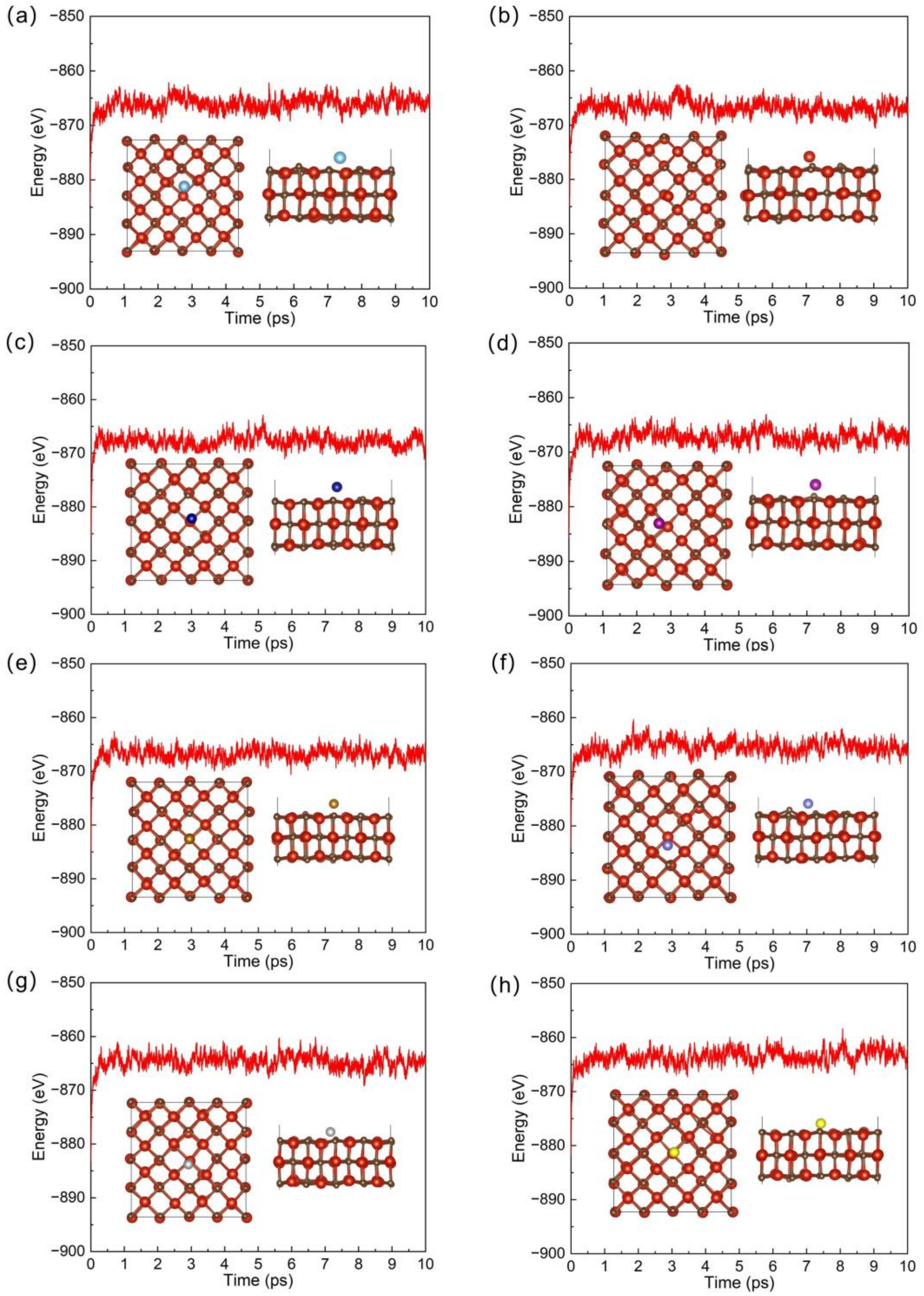

Figure S7. Energies as a function of time of (**a**) Ti@(4 × 4)-V_3C_3-$V_{surf\text{-}C}$, (**b**) V@(4 × 4)-V_3C_3-$V_{surf\text{-}C}$, (**c**) Cr@(4 × 4)-V_3C_3-$V_{surf\text{-}C}$, (**d**) Mn@(4 × 4)-V_3C_3-$V_{surf\text{-}C}$, (**e**) Fe@(4 × 4)-V_3C_3-$V_{surf\text{-}C}$, (**f**) Co@(4 × 4)-V_3C_3-$V_{surf\text{-}C}$, (**g**) Ni@(4 × 4)-V_3C_3-$V_{surf\text{-}C}$ and (**h**) Cu@(4 × 4)-V_3C_3-$V_{surf\text{-}C}$. during the AIMD simulations (inset: the configurations after 10 ps AIMD simulations).

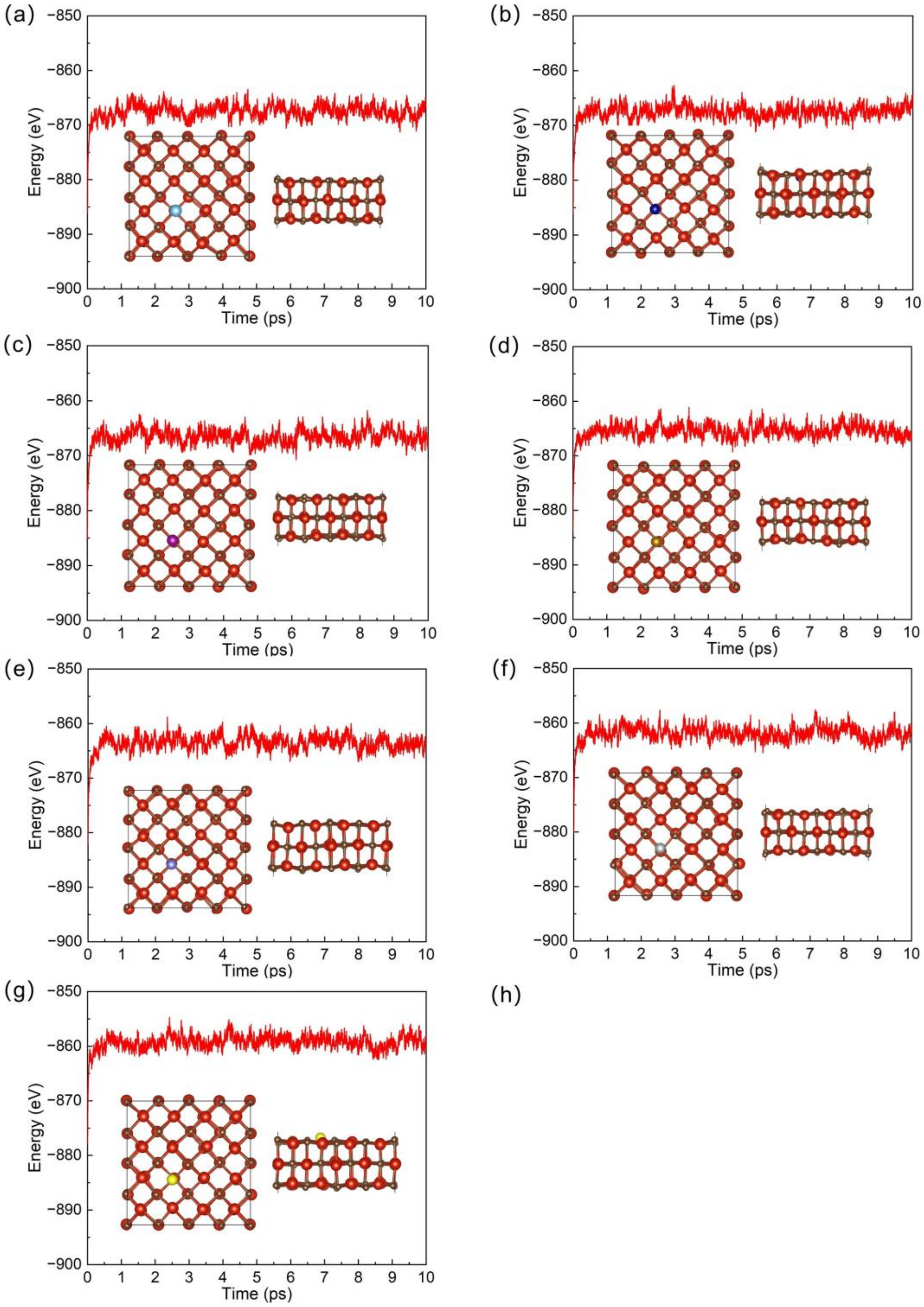

Figure S8. Energies as a function of time of (**a**) Ti@(4 × 4)-V_3C_3-$V_{surf\text{-}V}$, (**b**) Cr@(4 × 4)-V_3C_3-$V_{surf\text{-}V}$, (**c**) Mn@(4 × 4)-V_3C_3-$V_{surf\text{-}V}$, (**d**) Fe@(4 × 4)-V_3C_3-$V_{surf\text{-}V}$, (**e**) Co@(4 × 4)-V_3C_3-$V_{surf\text{-}V}$, (**f**) Ni@(4 × 4)-V_3C_3-$V_{surf\text{-}V}$ and (**g**) Cu@(4 × 4)-V_3C_3-$V_{surf\text{-}V}$. during the AIMD simulations (inset: the configurations after 10 ps AIMD simulations).

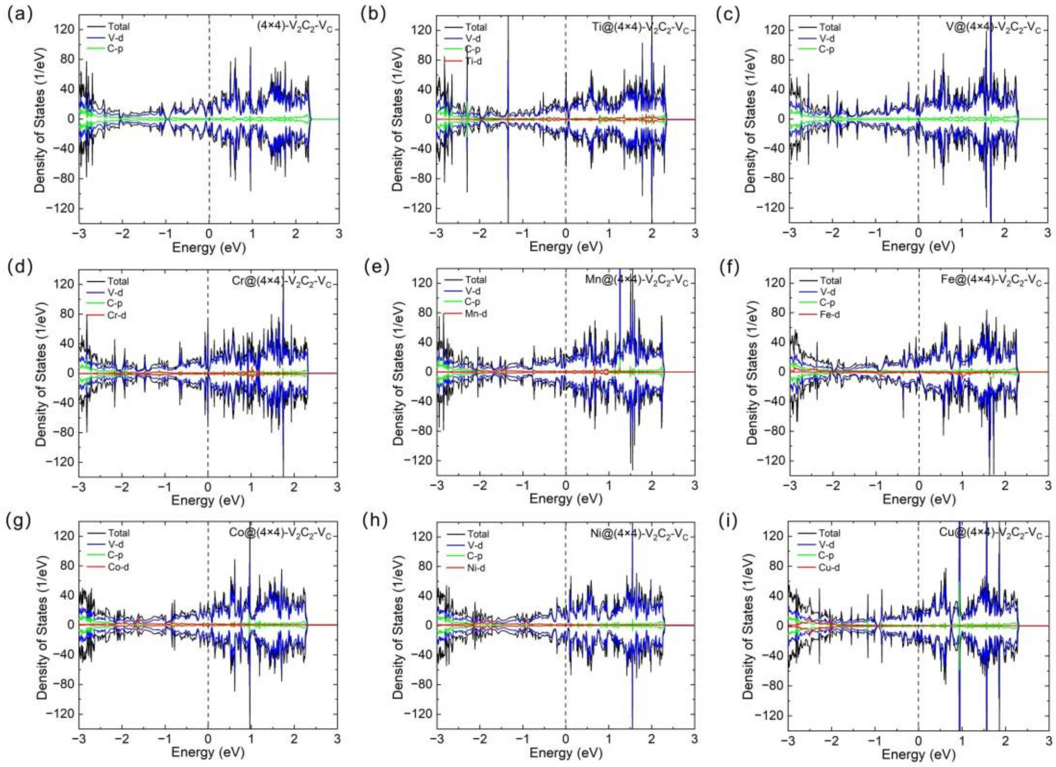

Figure S9. Total and partial density of states of (**a**) (4 × 4)-V_2C_2-V_C, (**b**) Ti@(4 × 4)-V_2C_2-V_C, (**c**) V@(4 × 4)-V_2C_2-V_C, (**d**) Cr@(4 × 4)-V_2C_2-V_C, (**e**) Mn@(4 × 4)-V_2C_2-V_C, (**f**) Fe@(4 × 4)-V_2C_2-V_C, (**g**) Co@(4 × 4)-V_2C_2-V_C, (**h**) Ni@(4 × 4)-V_2C_2-V_C and (**i**) Cu@(4 × 4)-V_2C_2-V_C. The Fermi level is set to zero and marked with the dashed line.

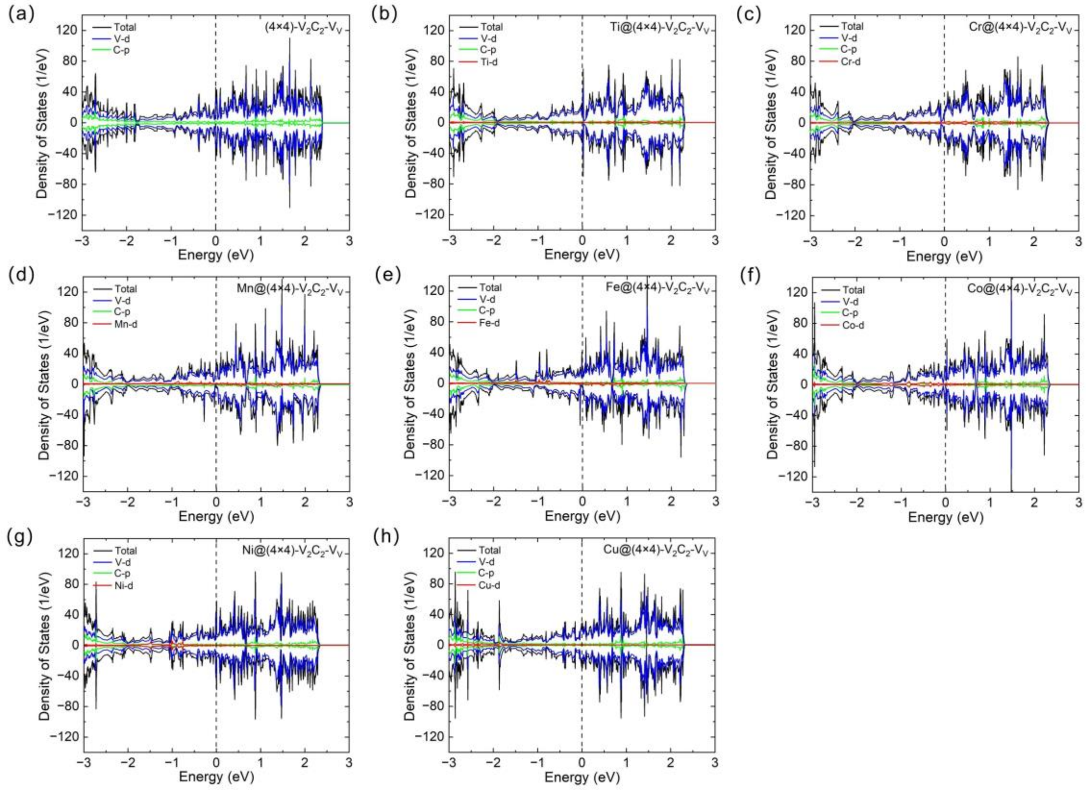

Figure S10. Total and partial density of states of (**a**) (4 × 4)-V_2C_2-V_V, (**b**) Ti@(4 × 4)-V_2C_2-V_V, (**c**) Cr@(4 × 4)-V_2C_2-V_V, (**d**) Mn@(4 × 4)-V_2C_2-V_V, (**e**) Fe@(4 × 4)-V_2C_2-V_V, (**f**) Co@(4 × 4)-V_2C_2-V_V, (**g**) Ni@(4 ×

4)-V_2C_2-V_V and (**h**) Cu@(4 × 4)-V_2C_2-V_V. The Fermi level is set to zero and marked with the dashed line.

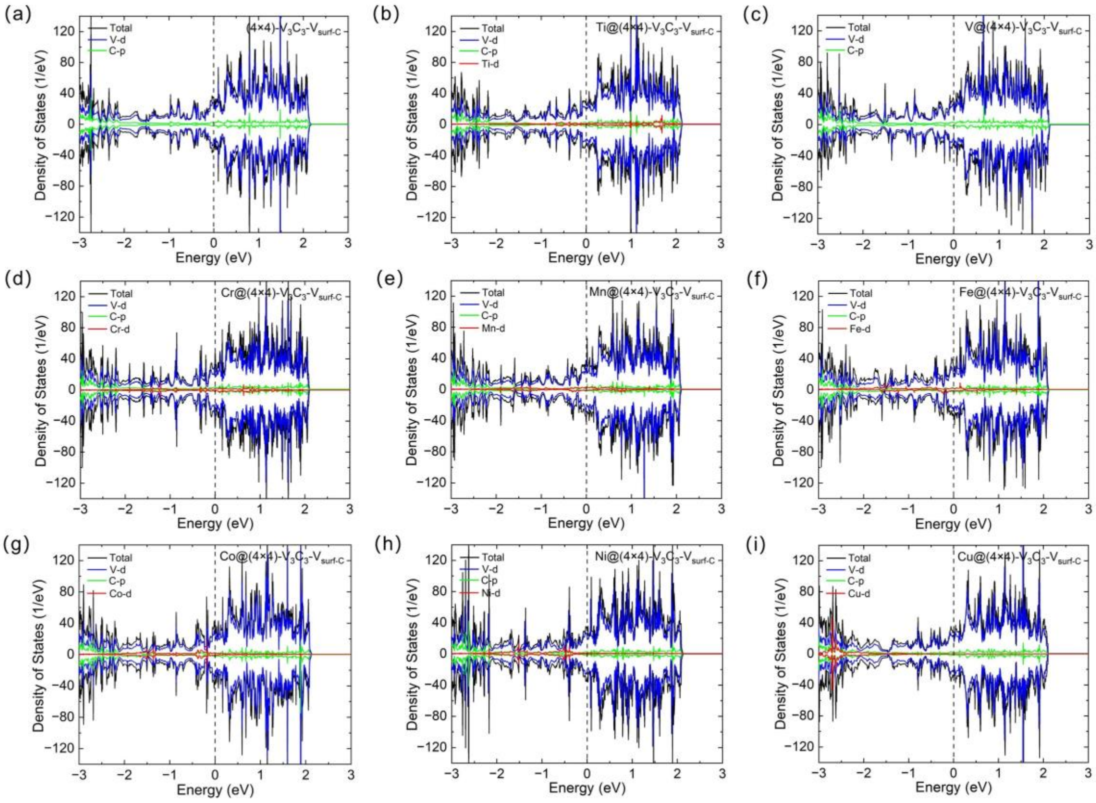

Figure S11. Total and partial density of states of (**a**) (4 × 4)-V_2C_2-V_{surf-C}, (**b**) Ti@(4 × 4)-V_2C_2-V_{surf-C}, (**c**) V@(4 × 4)-V_2C_2-V_{surf-C}, (**d**) Cr@(4 × 4)-V_2C_2-V_{surf-C}, (**e**) Mn@(4 × 4)-V_2C_2-V_{surf-C}, (**f**) Fe@(4 × 4)-V_2C_2-V_{surf-C}, (**g**) Co@(4 × 4)-V_2C_2-V_{surf-C}, (**h**) Ni@(4 × 4)-V_2C_2-V_{surf-C} and (**i**) Cu@(4 × 4)-V_2C_2-V_{surf-C}. The Fermi level is set to zero and marked with the dashed line.

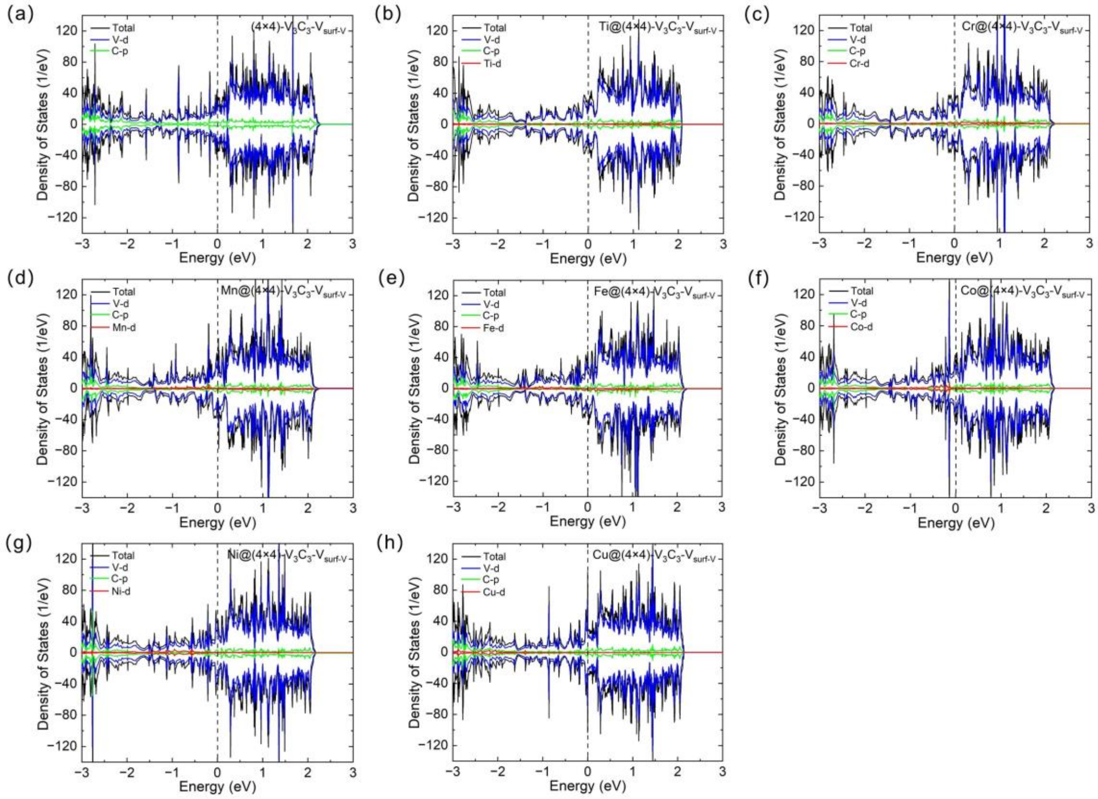

Figure S12. Total and partial density of states of (**a**) (4 × 4)-V_2C_2-V_{surf-V}, (**b**) Ti@(4 × 4)-V_2C_2-V_{surf-V}, (**c**) Cr@(4 × 4)-V_2C_2-V_{surf-V}, (**d**) Mn@(4 × 4)-V_2C_2-V_{surf-V}, (**e**) Fe@(4 × 4)-V_2C_2-V_{surf-V}, (**f**) Co@(4 × 4)-V_2C_2-V_{surf-V}, (**g**) Ni@(4 × 4)-V_2C_2-V_{surf-V} and (**h**) Cu@(4 × 4)-V_2C_2-V_{surf-V}. The Fermi level is set to zero and marked with the dashed line.

Table S1. The lattice constants (Å) of TM@(4 × 4)-V_2C_2-V_C.

Structure	*a* = *b*/Å
Ti@(4 × 4)-V_2C_2-V_C	11.415
V@(4 × 4)-V_2C_2-V_C	11.421
Cr@(4 × 4)-V_2C_2-V_C	11.428
Mn@(4 × 4)-V_2C_2-V_C	11.431
Fe@(4 × 4)-V_2C_2-V_C	11.419
Co@(4 × 4)-V_2C_2-V_C	11.431
Ni@(4 × 4)-V_2C_2-V_C	11.425
Cu@(4 × 4)-V_2C_2-V_C	11.408

Table S2. The lattice constants (Å) of TM@(4 × 4)-V_2C_2-V_V.

Structure	*a* = *b*/Å
Ti@(4 × 4)-V_2C_2-V_V	11.432
Cr@(4 × 4)-V_2C_2-V_V	11.404
Mn@(4 × 4)-V_2C_2-V_V	11.403
Fe@(4 × 4)-V_2C_2-V_V	11.400
Co@(4 × 4)-V_2C_2-V_V	11.394
Ni@(4 × 4)-V_2C_2-V_V	11.396
Cu@(4 × 4)-V_2C_2-V_V	11.402

Table S3. The lattice constants (Å) of TM@(4 × 4)-V_3C_3-V_{surf-C}.

Structure	*a* = *b*/Å
Ti@(4 × 4)-V_3C_3-V_{surf-C}	11.512
V@(4 × 4)-V_3C_3-V_{surf-C}	11.513
Cr@(4 × 4)-V_3C_3-V_{surf-C}	11.515
Mn@(4 × 4)-V_3C_3-V_{surf-C}	11.517
Fe@(4 × 4)-V_3C_3-V_{surf-C}	11.518
Co@(4 × 4)-V_3C_3-V_{surf-C}	11.522
Ni@(4 × 4)-V_3C_3-V_{surf-C}	11.517
Cu@(4 × 4)-V_3C_3-V_{surf-C}	11.516

Table S4. The lattice constants (Å) of TM@(4 × 4)-V_3C_3-V_{surf-V}.

Structure	*a* = *b*/Å
Ti@(4 × 4)-V_3C_3-V_{surf-V}	11.517
Cr@(4 × 4)-V_3C_3-V_{surf-V}	11.501
Mn@(4 × 4)-V_3C_3-V_{surf-V}	11.503
Fe@(4 × 4)-V_3C_3-V_{surf-V}	11.499
Co@(4 × 4)-V_3C_3-V_{surf-V}	11.496
Ni@(4 × 4)-V_3C_3-V_{surf-V}	11.495
Cu@(4 × 4)-V_3C_3-V_{surf-V}	11.497

Table S5. Calculated energies (*E*), zero-point energies (*E*zpe), vibrational entropy (TS_H), Gibbs free energies (*G*) and Gibbs free energy differences (Δ*G*) of Ti@(4 × 4)-V_2C_2-V_C at different adsorption sites.

	E/eV	E_{ZPE}/eV	TS_H/eV	*G*/eV	Δ*G*/eV
*+1/2H_2	−593.51	0.14	0.20	−593.57	0.00
*H(C^1)	−593.39	0.22	0.02	−593.19	0.38
*H(C^2)	−593.30	0.21	0.01	−593.10	0.47
*H(C^3)	−593.18	0.18	0.04	−593.04	0.53
*H(C^4)	−593.26	0.19	0.05	−593.12	0.45
*H(C^5)	−593.28	0.19	0.06	−593.15	0.42
*H(V^1)	−594.08	0.19	0.01	−593.90	−0.33
*H(V^2)	−593.19	0.16	0.02	−593.05	0.52
*H(V^3)	−593.18	0.16	0.02	−593.04	0.53
*H(Ti^1)	−593.31	0.11	0.07	−593.27	0.30

Table S6. Calculated energies (*E*), zero-point energies (*E*zpe), vibrational entropy (TS_H), Gibbs free energies (*G*) and Gibbs free energy differences (Δ*G*) of V@(4 × 4)-V_2C_2-V_C at different adsorption sites.

	E/eV	E_{ZPE}/eV	TS_H/eV	*G*/eV	Δ*G*/eV
*+1/2H_2	−594.13	0.14	0.20	−594.19	0.00
*H(C^1)	−593.96	0.22	0.02	−593.76	0.43
*H(C^2)	−593.94	0.22	0.02	−593.74	0.45
*H(C^3)	−593.83	0.18	0.03	−593.68	0.51
*H(C^4)	−593.86	0.19	0.05	−593.72	0.47
*H(C^5)	−593.90	0.19	0.05	−593.76	0.43
*H(V^1)	−594.59	0.18	0.01	−594.42	−0.23
*H(V^2)	−593.81	0.16	0.02	−593.67	0.52
*H(V^3)	−593.80	0.16	0.02	−593.66	0.53

*H($V^1_{imported}$)	−594.11	0.12	0.07	−594.06	0.13

Table S7. Calculated energies (*E*), zero-point energies (*E*zpe), vibrational entropy (TS_H), Gibbs free energies (*G*) and Gibbs free energy differences (Δ*G*) of Cr@(4 × 4)-V_2C_2-V_C at different adsorption sites.

	E/eV	E_{ZPE}/eV	TS_H/eV	*G*/eV	Δ*G*/eV
*+1/2H_2	−594.52	0.14	0.20	−594.58	0.00
*H(C^1)	−594.33	0.22	0.02	−594.13	0.45
*H(C^2)	−594.30	0.22	0.02	−594.10	0.48
*H(C^3)	−594.22	0.18	0.09	−594.13	0.45
*H(C^4)	−594.23	0.19	0.06	−594.10	0.48
*H(C^5)	−594.27	0.20	0.04	−594.11	0.47
*H(V^1)	−594.97	0.18	0.01	−594.80	−0.22
*H(V^2)	−594.19	0.16	0.02	−594.05	0.53
*H(V^3)	−594.19	0.16	0.02	−594.05	0.53
*H(Cr^1)	−594.72	0.13	0.05	−594.64	−0.06

Table S8. Calculated energies (*E*), zero-point energies (*E*zpe), vibrational entropy (TS_H), Gibbs free energies (*G*) and Gibbs free energy differences (Δ*G*) of Mn@(4 × 4)-V_2C_2-V_C at different adsorption sites.

	E/eV	E_{ZPE}/eV	TS_H/eV	*G*/eV	Δ*G*/eV
*+1/2H_2	−597.45	0.14	0.20	−594.13	0.00
*H(C^1)	−594.46	0.22	0.02	−594.26	−0.13
*H(C^2)	−594.33	0.21	0.01	−594.13	0.00
*H(C^3)	−594.23	0.19	0.07	−594.11	0.02
*H(C^4)	−594.32	0.20	0.05	−594.17	−0.04
*H(C^5)	−594.30	0.19	0.07	−594.18	−0.05
*H(V^1)	−595.09	0.19	0.01	−594.91	−0.78
*H(V^2)	−594.20	0.16	0.02	−594.06	0.07
*H(V^3)	−594.21	0.16	0.02	−594.07	0.06
*H(Mn^1)	−594.27	0.14	0.04	−594.17	−0.04

Table S9. Calculated energies (*E*), zero-point energies (*E*zpe), vibrational entropy (TS_H), Gibbs free energies (*G*) and Gibbs free energy differences (Δ*G*) of Fe@(4 × 4)-V_2C_2-V_C at different adsorption sites.

	E/eV	E_{ZPE}/eV	TS_H/eV	*G*/eV	Δ*G*/eV
*+1/2H_2	−593.91	0.14	0.20	−593.97	0.00
*H(C^1)	−593.77	0.22	0.02	−593.57	0.40
*H(C^2)	−593.67	0.21	0.02	−593.48	0.49
*H(C^3)	−593.61	0.19	0.06	−593.48	0.49
*H(C^4)	−593.67	0.20	0.05	−593.52	0.45
*H(C^5)	−593.67	0.19	0.06	−593.54	0.43
*H(V^1)	−594.28	0.18	0.01	−594.11	−0.14
*H(V^2)	−593.57	0.16	0.02	−593.43	0.54
*H(V^3)	−593.58	0.16	0.02	−593.44	0.53
*H(Fe^1)	−594.02	0.14	0.05	−593.93	0.04

Table S10. Calculated energies (*E*), zero-point energies (*E*zpe), vibrational entropy (TS_H), Gibbs free energies (*G*) and Gibbs free energy differences (Δ*G*) of Co@(4 × 4)-V_2C_2-V_C at different adsorption sites.

	E/eV	E_{ZPE}/eV	TS_H/eV	*G*/eV	Δ*G*/eV
*+1/2H_2	−592.65	0.14	0.20	−592.71	0.00
*H(C^1)	−592.55	0.21	0.02	−592.36	0.35
*H(C^2)	−592.44	0.18	0.02	−592.28	0.43
*H(C^3)	−592.41	0.19	0.06	−592.28	0.43
*H(C^4)	−592.45	0.20	0.05	−592.30	0.41
*H(C^5)	−592.44	0.19	0.06	−592.31	0.40
*H(V^1)	−593.10	0.17	0.01	−592.94	−0.23
*H(V^2)	−592.35	0.16	0.02	−592.21	0.50
*H(V^3)	−592.37	0.16	0.02	−592.23	0.48
*H(Co^1)	−593.05	0.15	0.04	−592.94	−0.23

Table S11. Calculated energies (*E*), zero-point energies (*E*zpe), vibrational entropy (TS_H), Gibbs free energies (*G*) and Gibbs free energy differences (Δ*G*) of Ni@(4 × 4)-V_2C_2-V_C at different adsorption sites.

	E/eV	E_{ZPE}/eV	TS_H/eV	*G*/eV	Δ*G*/eV
*+1/2H_2	−591.63	0.14	0.20	−591.69	0.00
*H(C^1)	−591.49	0.21	0.02	−591.30	0.39
*H(C^2)	−591.37	0.20	0.05	−591.22	0.47
*H(C^3)	−591.34	0.19	0.06	−591.21	0.48
*H(C^4)	−591.39	0.20	0.04	−591.23	0.46
*H(C^5)	−591.35	0.18	0.11	−591.28	0.41
*H(V^1)	−592.03	0.17	0.01	−591.87	−0.18
*H(V^2)	−591.27	0.16	0.02	−591.13	0.56
*H(V^3)	−591.31	0.16	0.02	−591.17	0.52
*H(Ni^1)	−591.99	0.15	0.04	−591.88	−0.19

Table S12. Calculated energies (*E*), zero-point energies (*E*zpe), vibrational entropy (TS_H), Gibbs free energies (*G*) and Gibbs free energy differences (Δ*G*) of Cu@(4 × 4)-V_2C_2-V_C at different adsorption sites.

	E/eV	E_{ZPE}/eV	TS_H/eV	*G*/eV	Δ*G*/eV
*+1/2H_2	−590.19	0.14	0.20	−590.25	0.00
*H(C^1)	−590.08	0.21	0.03	−589.90	0.35
*H(C^2)	−589.93	0.21	0.02	−589.74	0.51
*H(C^3)	−589.90	0.19	0.05	−589.76	0.49
*H(C^4)	−589.98	0.20	0.05	−589.83	0.42
*H(C^5)	−589.93	0.18	0.08	−589.83	0.42
*H(V^1)	−590.46	0.18	0.01	−590.29	−0.04
*H(V^2)	−589.86	0.16	0.02	−589.72	0.53
*H(V^3)	−589.88	0.16	0.02	−589.74	0.51
*H(Cu^1)	−590.15	0.14	0.05	−590.06	0.19

Table S13. Calculated energies (E), zero-point energies (Ezpe), vibrational entropy (TS_H), Gibbs free energies (G) and Gibbs free energy differences (ΔG) of Ti@(4 × 4)-V_2C_2-V_V at different adsorption sites.

	E/eV	E_{ZPE}/eV	TS_H/eV	G/eV	ΔG/eV
*+1/2H_2	−595.97	0.14	0.20	−596.03	0.00
*H(C^1)	−595.77	0.21	0.04	−595.60	0.43
*H(C^2)	−595.70	0.19	0.06	−595.57	0.46
*H(C^3)	−595.72	0.20	0.04	−595.56	0.47
*H(V^1)	−595.65	0.16	0.02	−595.51	0.52
*H(V^2)	−595.63	0.16	0.02	−595.49	0.54
*H(V^3)	−595.65	0.16	0.02	−595.51	0.52
*H(V^4)	−595.65	0.16	0.02	−595.51	0.52
*H(V^5)	−595.66	0.16	0.02	−595.52	0.51
*H(Ti^1)	−595.36	0.14	0.03	−595.25	0.78

Table S14. Calculated energies (E), zero-point energies (Ezpe), vibrational entropy (TS_H), Gibbs free energies (G) and Gibbs free energy differences (ΔG) of Cr@(4 × 4)-V_2C_2-V_V at different adsorption sites.

	E/eV	E_{ZPE}/eV	TS_H/eV	G/eV	ΔG/eV
*+1/2H_2	−596.43	0.14	0.20	−596.49	0.00
*H(C^1)	−596.19	0.20	0.04	−596.03	0.46
*H(C^2)	−596.16	0.19	0.06	−596.03	0.46
*H(C^3)	−596.15	0.19	0.06	−596.02	0.47
*H(V^1)	−596.12	0.16	0.02	−595.98	0.51
*H(V^2)	−596.13	0.16	0.02	−595.99	0.50
*H(V^3)	−596.11	0.16	0.02	−595.97	0.52
*H(V^4)	−596.13	0.16	0.02	−595.99	0.50
*H(V^5)	−596.12	0.16	0.02	−595.98	0.51
*H(Cr^1)	−596.36	0.19	0.01	−596.18	0.31

Table S15. Calculated energies (E), zero-point energies (Ezpe), vibrational entropy (TS_H), Gibbs free energies (G) and Gibbs free energy differences (ΔG) of Mn@(4 × 4)-V_2C_2-V_V at different adsorption sites.

	E/eV	E_{ZPE}/eV	TS_H/eV	G/eV	ΔG/eV
*+1/2H_2	−595.34	0.14	0.20	−595.40	0.00
*H(C^1)	−595.17	0.20	0.01	−594.98	0.42
*H(C^2)	−595.08	0.19	0.06	−594.95	0.45
*H(C^3)	−595.07	0.19	0.05	−594.93	0.47
*H(V^1)	−594.97	0.16	0.02	−594.83	0.57
*H(V^2)	−595.04	0.16	0.02	−594.90	0.50
*H(V^3)	−595.03	0.16	0.02	−594.89	0.51
*H(V^4)	−595.03	0.16	0.02	−594.89	0.51
*H(V^5)	−595.01	0.16	0.02	−594.87	0.53
*H(Mn^1)	−595.28	0.20	0.01	−595.09	0.31

Table S16. Calculated energies (*E*), zero-point energies (*E*zpe), vibrational entropy (TS_H), Gibbs free energies (*G*) and Gibbs free energy differences (Δ*G*) of Fe@(4 × 4)-V_2C_2-V_V at different adsorption sites.

	***E*/eV**	**E_{ZPE}/eV**	**TS_H/eV**	***G*/eV**	**Δ*G*/eV**
*+1/2H_2	−594.08	0.14	0.20	−594.14	0.00
*H(C^1)	−593.95	0.20	0.01	−593.76	0.38
*H(C^2)	−593.82	0.19	0.05	−593.68	0.46
*H(C^3)	−593.81	0.21	0.03	−593.63	0.51
*H(V^1)	−593.69	0.16	0.02	−593.55	0.59
*H(V^2)	−593.79	0.17	0.02	−593.64	0.50
*H(V^3)	−593.75	0.16	0.02	−593.61	0.53
*H(V^4)	−593.78	0.16	0.02	−593.64	0.50
*H(V^5)	−593.75	0.17	0.02	−593.60	0.54
*H(Fe^1)	−593.98	0.20	0.01	−593.79	0.35

Table S17. Calculated energies (*E*), zero-point energies (*E*zpe), vibrational entropy (TS_H), Gibbs free energies (*G*) and Gibbs free energy differences (Δ*G*) of Co@(4 × 4)-V_2C_2-V_V at different adsorption sites.

	***E*/eV**	**E_{ZPE}/eV**	**TS_H/eV**	***G*/eV**	**Δ*G*/eV**
*+1/2H_2	−592.55	0.14	0.20	−592.61	0.00
*H(C^1)	−592.47	0.20	0.01	−592.28	0.33
*H(C^2)	−592.29	0.21	0.02	−592.10	0.51
*H(C^3)	−592.25	0.21	0.02	−592.06	0.55
*H(V^1)	−592.08	0.16	0.02	−591.94	0.67
*H(V^2)	−592.22	0.17	0.02	−592.07	0.54
*H(V^3)	−592.17	0.16	0.02	−592.03	0.58
*H(V^4)	−592.20	0.17	0.02	−592.05	0.56
*H(V^5)	−592.16	0.17	0.02	−592.01	0.60
*H(Co^1)	−592.33	0.20	0.01	−592.14	0.47

Table S18. Calculated energies (*E*), zero-point energies (*E*zpe), vibrational entropy (TS_H), Gibbs free energies (*G*) and Gibbs free energy differences (Δ*G*) of Ni@(4 × 4)-V_2C_2-V_V at different adsorption sites.

	***E*/eV**	**E_{ZPE}/eV**	**TS_H/eV**	***G*/eV**	**Δ*G*/eV**
*+1/2H_2	−590.71	0.14	0.20	−590.77	0.00
*H(C^1)	−590.62	0.20	0.01	−590.43	0.34
*H(C^2)	−590.46	0.20	0.05	−590.31	0.46
*H(C^3)	−590.43	0.21	0.02	−590.24	0.53
*H(V^1)	−590.24	0.16	0.02	−590.10	0.67
*H(V^2)	−590.41	0.17	0.02	−590.26	0.51
*H(V^3)	−590.34	0.16	0.02	−590.20	0.57
*H(V^4)	−590.40	0.17	0.02	−590.25	0.52
*H(V^5)	−590.35	0.17	0.02	−590.20	0.57
*H(Ni^1)	−590.02	0.18	0.02	−589.86	0.91

Table S19. Calculated energies (*E*), zero-point energies (*E*zpe), vibrational entropy (TS_H), Gibbs free energies (*G*) and Gibbs free energy differences (Δ*G*) of Cu@(4 × 4)-V_2C_2-V_V at different adsorption sites.

	E/eV	E_{ZPE}/eV	TS_H/eV	*G*/eV	Δ*G*/eV
*+1/2H_2	−588.08	0.14	0.20	−588.14	0.00
*H(C^1)	−587.89	0.20	0.01	−587.70	0.44
*H(C^2)	−587.83	0.20	0.04	−587.67	0.47
*H(C^3)	−587.76	0.19	0.06	−587.63	0.51
*H(V^1)	−587.65	0.16	0.02	−587.51	0.63
*H(V^2)	−587.79	0.17	0.02	−587.64	0.50
*H(V^3)	−587.66	0.16	0.02	−587.52	0.62
*H(V^4)	−587.76	0.16	0.02	−587.62	0.52
*H(V^5)	−587.73	0.17	0.02	−587.58	0.56
*H(Cu^1)	−586.77	0.14	0.03	−586.66	1.48

Table S20. Calculated energies (*E*), zero-point energies (*E*zpe), vibrational entropy (TS_H), Gibbs free energies (*G*) and Gibbs free energy differences (Δ*G*) of Ti@(4 × 4)-V_3C_3-$V_{surf\text{-}C}$ at different adsorption sites.

	E/eV	E_{ZPE}/eV	TS_H/eV	*G*/eV	Δ*G*/eV
*+1/2H_2	−908.65	0.14	0.20	−908.71	0.00
*H(C^1)	−909.10	0.24	0.01	−908.87	−0.16
*H(C^2)	−908.75	0.24	0.01	−908.52	0.19
*H(C^3)	−908.77	0.24	0.01	−908.54	0.17
*H(C^4)	−908.85	0.24	0.01	−908.62	0.09
*H(C^5)	−908.92	0.24	0.01	−908.69	0.02
*H(V^1)	−909.10	0.19	0.01	−908.92	−0.21
*H(V^2)	−908.20	0.17	0.02	−908.05	0.66
*H(V^3)	−908.20	0.17	0.02	−908.05	0.66
*H(Ti^1)	−908.56	0.11	0.08	−908.53	0.18

Table S21. Calculated energies (*E*), zero-point energies (*E*zpe), vibrational entropy (TS_H), Gibbs free energies (*G*) and Gibbs free energy differences (Δ*G*) of V@(4 × 4)-V_3C_3-$V_{surf\text{-}C}$ at different adsorption sites.

	E/eV	E_{ZPE}/eV	TS_H/eV	*G*/eV	Δ*G*/eV
*+1/2H_2	−908.97	0.14	0.20	−909.03	0.00
*H(C^1)	−909.62	0.24	0.01	−909.39	−0.36
*H(C^2)	−909.13	0.24	0.01	−908.90	0.13
*H(C^3)	−909.09	0.24	0.01	−908.86	0.17
*H(C^4)	−909.17	0.24	0.01	−908.94	0.09
*H(C^5)	−909.23	0.24	0.01	−909.00	0.03
*H(V^1)	−909.42	0.19	0.01	−909.24	−0.21
*H(V^2)	−908.51	0.17	0.02	−908.37	0.66
*H(V^3)	−908.51	0.17	0.02	−908.37	0.66
*H($V^1_{imported}$)	−909.04	0.12	0.06	−908.98	0.05

Table S22. Calculated energies (E), zero-point energies (Ezpe), vibrational entropy (TS_H), Gibbs free energies (G) and Gibbs free energy differences (ΔG) of Cr@(4 × 4)-V_3C_3-V_{surf-C} at different adsorption sites.

	E/eV	E_{ZPE}/eV	TS_H/eV	G/eV	ΔG/eV
*+1/2H_2	−909.13	0.14	0.20	−909.19	0.00
*H(C^1)	−910.34	0.25	0.01	−910.10	−0.91
*H(C^2)	−910.11	0.23	0.01	−909.89	−0.70
*H(C^3)	−910.19	0.24	0.01	−909.96	−0.77
*H(C^4)	−910.29	0.24	0.01	−910.06	−0.87
*H(C^5)	−910.30	0.24	0.01	−910.07	−0.88
*H(V^1)	−910.50	0.18	0.01	−910.33	−1.14
*H(V^2)	−909.64	0.17	0.02	−909.49	−0.30
*H(V^3)	−909.66	0.17	0.02	−909.51	−0.32
*H(Cr^1)	−909.39	0.15	0.04	−909.28	−0.09

Table S23. Calculated energies (E), zero-point energies (Ezpe), vibrational entropy (TS_H), Gibbs free energies (G) and Gibbs free energy differences (ΔG) of Mn@(4 × 4)-V_3C_3-V_{surf-C} at different adsorption sites.

	E/eV	E_{ZPE}/eV	TS_H/eV	G/eV	ΔG/eV
*+1/2H_2	−908.64	0.14	0.20	−908.70	0.00
*H(C^1)	−910.17	0.25	0.01	−909.93	−1.23
*H(C^2)	−909.97	0.24	0.01	−909.74	−1.04
*H(C^3)	−910.03	0.24	0.01	−909.80	−1.10
*H(C^4)	−910.11	0.24	0.01	−909.88	−1.18
*H(C^5)	−910.13	0.24	0.01	−909.90	−1.20
*H(V^1)	−910.34	0.19	0.01	−910.16	−1.46
*H(V^2)	−909.47	0.17	0.02	−909.32	−0.62
*H(V^3)	−909.48	0.17	0.02	−909.33	−0.63
*H(Mn^1)	−909.82	0.12	0.07	−909.77	−1.07

Table S24. Calculated energies (E), zero-point energies (Ezpe), vibrational entropy (TS_H), Gibbs free energies (G) and Gibbs free energy differences (ΔG) of Fe@(4 × 4)-V_3C_3-V_{surf-C} at different adsorption sites.

	E/eV	E_{ZPE}/eV	TS_H/eV	G/eV	ΔG/eV
*+1/2H_2	−908.25	0.14	0.20	−908.31	0.00
*H(C^1)	−909.26	0.25	0.01	−909.02	−0.71
*H(C^2)	−909.17	0.24	0.01	−908.94	−0.63
*H(C^3)	−909.18	0.24	0.01	−908.95	−0.64
*H(C^4)	−909.27	0.24	0.01	−909.04	−0.73
*H(C^5)	−909.29	0.24	0.01	−909.06	−0.75
*H(V^1)	−909.37	0.18	0.01	−909.20	−0.89
*H(V^2)	−908.60	0.17	0.02	−908.45	−0.14
*H(V^3)	−908.62	0.17	0.02	−908.47	−0.16
*H(Fe^1)	−908.44	0.16	0.04	−908.32	−0.01

Table S25. Calculated energies (E), zero-point energies (Ezpe), vibrational entropy (TS_H), Gibbs free energies (G) and Gibbs free energy differences (ΔG) of Co@(4 × 4)-V_3C_3-$V_{surf\text{-}C}$ at different adsorption sites.

	E/eV	E_{ZPE}/eV	TS_H/eV	G/eV	ΔG/eV
*+1/2H_2	−907.62	0.14	0.20	−907.68	0.00
*H(C^1)	−908.06	0.25	0.01	−907.82	−0.14
*H(C^2)	−907.89	0.24	0.01	−907.66	0.02
*H(C^3)	−907.89	0.24	0.01	−907.66	0.02
*H(C^4)	−907.97	0.24	0.01	−907.74	−0.06
*H(C^5)	−907.85	0.24	0.01	−907.62	0.06
*H(V^1)	−908.08	0.18	0.01	−907.91	−0.23
*H(V^2)	−907.32	0.17	0.02	−907.17	0.51
*H(V^3)	−907.34	0.17	0.02	−907.19	0.49
*H(Co^1)	−907.87	0.15	0.04	−907.76	−0.08

Table S26. Calculated energies (E), zero-point energies (Ezpe), vibrational entropy (TS_H), Gibbs free energies (G) and Gibbs free energy differences (ΔG) of Ni@(4 × 4)-V_3C_3-$V_{surf\text{-}C}$ at different adsorption sites.

	E/eV	E_{ZPE}/eV	TS_H/eV	G/eV	ΔG/eV
*+1/2H_2	−906.94	0.14	0.20	−907.00	0.00
*H(C^1)	−907.09	0.25	0.01	−906.85	0.15
*H(C^2)	−907.02	0.24	0.01	−906.79	0.21
*H(C^3)	−907.04	0.24	0.01	−906.81	0.19
*H(C^4)	−907.13	0.24	0.01	−906.90	0.10
*H(C^5)	−907.14	0.24	0.01	−906.91	0.09
*H(V^1)	−907.20	0.18	0.01	−907.03	−0.03
*H(V^2)	−906.47	0.17	0.02	−906.32	0.68
*H(V^3)	−906.49	0.17	0.02	−906.34	0.66
*H(Ni^1)	−906.99	0.14	0.05	−906.90	0.10

Table S27. Calculated energies (E), zero-point energies (Ezpe), vibrational entropy (TS_H), Gibbs free energies (G) and Gibbs free energy differences (ΔG) of Cu@(4 × 4)-V_3C_3-$V_{surf\text{-}C}$ at different adsorption sites.

	E/eV	E_{ZPE}/eV	TS_H/eV	G/eV	ΔG/eV
*+1/2H_2	−905.58	0.14	0.20	−905.64	0.00
*H(C^1)	−905.65	0.25	0.01	−905.41	0.23
*H(C^2)	−905.59	0.24	0.01	−905.36	0.28
*H(C^3)	−905.66	0.24	0.01	−905.43	0.21
*H(C^4)	−905.75	0.24	0.01	−905.52	0.12
*H(C^5)	−905.76	0.24	0.01	−905.53	0.11
*H(V^1)	−905.77	0.18	0.01	−905.60	0.04
*H(V^2)	−905.11	0.17	0.02	−904.96	0.68
*H(V^3)	−905.13	0.17	0.02	−904.98	0.66
*H(Cu^1)	−905.29	0.13	0.07	−905.23	0.41

Table S28. Calculated energies (E), zero-point energies (E_{ZPE}), vibrational entropy (TS_H), Gibbs free energies (G) and Gibbs free energy differences (ΔG) of Ti@(4 × 4)-V_3C_3-$V_{surf\text{-}V}$ at different adsorption sites.

	E/eV	E_{ZPE}/eV	TS_H/eV	G/eV	ΔG/eV
*+1/2H_2	−910.23	0.14	0.20	−910.29	0.00
*H(C^1)	−910.37	0.24	0.01	−910.14	0.15
*H(C^2)	−910.40	0.24	0.01	−910.17	0.12
*H(C^3)	−910.45	0.24	0.01	−910.22	0.07
*H(V^1)	−909.81	0.17	0.02	−909.66	0.63
*H(V^2)	−909.76	0.17	0.02	−909.61	0.68
*H(V^3)	−909.81	0.17	0.02	−909.66	0.63
*H(V^4)	−909.81	0.17	0.02	−909.66	0.63
*H(V^5)	−909.81	0.17	0.02	−909.66	0.63
*H(Ti^1)	−909.46	0.14	0.03	−909.35	0.94

Table S29. Calculated energies (E), zero-point energies (E_{ZPE}), vibrational entropy (TS_H), Gibbs free energies (G) and Gibbs free energy differences (ΔG) of Cr@(4 × 4)-V_3C_3-$V_{surf\text{-}V}$ at different adsorption sites.

	E/eV	E_{ZPE}/eV	TS_H/eV	G/eV	ΔG/eV
*+1/2H_2	−910.19	0.14	0.20	−910.25	0.00
*H(C^1)	−910.53	0.25	0.01	−910.29	−0.04
*H(C^2)	−910.44	0.24	0.01	−910.21	0.04
*H(C^3)	−910.40	0.24	0.01	−910.17	0.08
*H(V^1)	−909.75	0.17	0.02	−909.60	0.65
*H(V^2)	−909.79	0.17	0.02	−909.64	0.61
*H(V^3)	−909.76	0.17	0.02	−909.61	0.64
*H(V^4)	−909.77	0.17	0.02	−909.62	0.63
*H(V^5)	−909.77	0.17	0.02	−909.62	0.63
*H(Cr^1)	−909.98	0.19	0.01	−909.80	0.45

Table S30. Calculated energies (E), zero-point energies (E_{ZPE}), vibrational entropy (TS_H), Gibbs free energies (G) and Gibbs free energy differences (ΔG) of Mn@(4 × 4)-V_3C_3-$V_{surf\text{-}V}$ at different adsorption sites.

	E/eV	E_{ZPE}/eV	TS_H/eV	G/eV	ΔG/eV
*+1/2H_2	−909.07	0.14	0.20	−909.13	0.00
*H(C^1)	−909.42	0.25	0.01	−909.18	−0.05
*H(C^2)	−909.28	0.24	0.01	−909.05	0.08
*H(C^3)	−909.26	0.24	0.01	−909.03	0.10
*H(V^1)	−908.63	0.17	0.02	−908.48	0.65
*H(V^2)	−908.63	0.17	0.02	−908.48	0.65
*H(V^3)	−908.63	0.17	0.02	−908.48	0.65
*H(V^4)	−908.64	0.17	0.02	−908.49	0.64
*H(V^5)	−908.65	0.17	0.02	−908.50	0.63
*H(Mn^1)	−908.74	0.19	0.01	−908.56	0.57

Table S31. Calculated energies (E), zero-point energies (E_{zpe}), vibrational entropy (TS_H), Gibbs free energies (G) and Gibbs free energy differences (ΔG) of Fe@(4 × 4)-V_3C_3-$V_{surf\text{-}V}$ at different adsorption sites.

	E/eV	E_{ZPE}/eV	TS_H/eV	G/eV	ΔG/eV
*+1/2H_2	−907.72	0.14	0.20	−907.78	0.00
*H(C^1)	−908.14	0.25	0.01	−907.90	−0.12
*H(C^2)	−907.93	0.24	0.01	−907.70	0.08
*H(C^3)	−907.89	0.24	0.01	−907.66	0.12
*H(V^1)	−907.27	0.17	0.02	−907.12	0.66
*H(V^2)	−907.28	0.17	0.02	−907.13	0.65
*H(V^3)	−907.28	0.17	0.02	−907.13	0.65
*H(V^4)	−907.29	0.17	0.02	−907.14	0.64
*H(V^5)	−907.30	0.17	0.02	−907.15	0.63
*H(Fe^1)	−907.38	0.20	0.01	−907.19	0.59

Table S32. Calculated energies (E), zero-point energies (E_{zpe}), vibrational entropy (TS_H), Gibbs free energies (G) and Gibbs free energy differences (ΔG) of Co@(4 × 4)-V_3C_3-$V_{surf\text{-}V}$ at different adsorption sites.

	E/eV	E_{ZPE}/eV	TS_H/eV	G/eV	ΔG/eV
*+1/2H_2	−906.05	0.14	0.20	−906.11	0.00
*H(C^1)	−906.60	0.25	0.01	−906.36	−0.25
*H(C^2)	−906.31	0.24	0.01	−906.08	0.03
*H(C^3)	−906.26	0.24	0.01	−906.03	0.08
*H(V^1)	−905.62	0.17	0.02	−905.47	0.64
*H(V^2)	−905.65	0.17	0.02	−905.50	0.61
*H(V^3)	−905.60	0.17	0.02	−905.45	0.66
*H(V^4)	−905.65	0.17	0.02	−905.50	0.61
*H(V^5)	−905.63	0.17	0.02	−905.48	0.63
*H(Co^1)	−905.70	0.20	0.01	−905.51	0.60

Table S33. Calculated energies (E), zero-point energies (E_{zpe}), vibrational entropy (TS_H), Gibbs free energies (G) and Gibbs free energy differences (ΔG) of Ni@(4 × 4)-V_3C_3-$V_{surf\text{-}V}$ at different adsorption sites.

	E/eV	E_{ZPE}/eV	TS_H/eV	G/eV	ΔG/eV
*+1/2H_2	−904.27	0.14	0.20	−904.33	0.00
*H(C^1)	−904.75	0.25	0.01	−905.51	−0.18
*H(C^2)	−904.48	0.24	0.01	−904.25	0.08
*H(C^3)	−904.43	0.24	0.01	−904.20	0.13
*H(V^1)	−903.82	0.17	0.02	−903.67	0.66
*H(V^2)	−903.83	0.17	0.02	−903.68	0.65
*H(V^3)	−903.81	0.17	0.02	−903.66	0.67
*H(V^4)	−903.84	0.17	0.02	−903.69	0.64
*H(V^5)	−903.86	0.17	0.02	−903.71	0.62
*H(Ni^1)	−903.51	0.18	0.02	−903.35	0.98

Table S34. Calculated energies (E), zero-point energies (E_{ZPE}), vibrational entropy (TS_H), Gibbs free energies (G) and Gibbs free energy differences (ΔG) of Cu@(4 × 4)-V_3C_3-$V_{surf\text{-}V}$ at different adsorption sites.

	E/eV	E_{ZPE}/eV	TS_H/eV	G/eV	ΔG/eV
*+1/2H_2	−901.78	0.14	0.20	−901.84	0.00
*H(C^1)	−902.12	0.25	0.01	−901.88	−0.04
*H(C^2)	−901.97	0.24	0.01	−901.74	0.10
*H(C^3)	−901.97	0.24	0.01	−901.74	0.10
*H(V^1)	−901.35	0.17	0.02	−901.20	0.64
*H(V^2)	−901.32	0.17	0.02	−901.17	0.67
*H(V^3)	−901.32	0.17	0.02	−901.17	0.67
*H(V^4)	−901.34	0.17	0.02	−901.19	0.65
*H(V^5)	−901.36	0.17	0.02	−901.21	0.63
*H(Cu^1)	−900.56	0.13	0.04	−900.47	1.37
